# SCA-1 micro-heterogeneity in the fate decision of dystrophic fibro/adipogenic progenitors

**DOI:** 10.1038/s41419-021-03408-1

**Published:** 2021-01-25

**Authors:** Giulio Giuliani, Simone Vumbaca, Claudia Fuoco, Cesare Gargioli, Ezio Giorda, Giorgia Massacci, Alessandro Palma, Alessio Reggio, Federica Riccio, Marco Rosina, Maria Vinci, Luisa Castagnoli, Gianni Cesareni

**Affiliations:** 1 Department of Biology, University of Rome “Tor Vergata”, Rome, Italy; 2 Core Facilities, Bambino Gesù Children’s Hospital – IRCCS, Rome, Italy; 3 Department of Onco-haematology, Cell and Gene Therapy, Bambino Gesù Children’s Hospital – IRCCS, Rome, Italy; 4 Fondazione Santa Lucia Istituto di Ricovero e Cura a Carattere Scientifico (IRCCS), Rome, Italy

**Keywords:** Cell biology, Mesenchymal stem cells

## Abstract

The term micro-heterogeneity refers to non-genetic cell to cell variability observed in a bell-shaped distribution of the expression of a trait within a population. The contribution of micro-heterogeneity to physiology and pathology remains largely uncharacterised. To address such an issue, we investigated the impact of heterogeneity in skeletal muscle fibro/adipogenic progenitors (FAPs) isolated from an animal model of Duchenne muscular dystrophy (DMD), the *mdx* mouse. FAPs play an essential role in muscle homoeostasis. However, in pathological conditions or ageing, they are the source of intramuscular infiltrations of fibrotic or adipose tissue. By applying a multiplex flow cytometry assay, we characterised and purified from *mdx* muscles two FAP cell states expressing different levels of SCA-1. The two cell states are morphologically identical and repopulate each other after several growth cycles. However, they differ in their in vitro behaviour. Cells expressing higher levels of SCA-1 (SCA1-High-FAPs) differentiate more readily into adipocytes while, when exposed to a fibrogenic stimulation, increase the expression of *Col1a1* and *Timp1* mRNA. A transcriptomic analysis confirmed the adipogenic propensity of SCA1-High-FAPs. In addition, SCA1-High-FAPs proliferate more extensively ex vivo and display more proliferating cells in dystrophic muscles in comparison to SCA1-Low-FAPs. Adipogenesis of both FAP cell states is inhibited in vitro by leucocytes from young dystrophic mice, while leucocytes isolated from aged dystrophic mice are less effective in limiting the adipogenesis of SCA1-High-FAPs suggesting a differential regulatory effect of the microenvironment on micro-heterogeneity. Our data suggest that FAP micro-heterogeneity is modulated in pathological conditions and that this heterogeneity in turn may impact on the behaviour of interstitial mesenchymal cells in genetic diseases.

## Introduction

The human body is estimated to contain approximately 200 different cell types^1^. However, this estimate relies on the experimental tools used to investigate cell populations and to define cell types. Developments in single-cell technologies have challenged this view revealing that cell populations often display a higher molecular heterogeneity than originally reported^2–4^. This new perspective raises several fundamental questions regarding the origin of cell phenotypic or molecular heterogeneity and its impact on physiological and pathological processes^5^. Two different types of cell heterogeneities may be distinguished, macro-heterogeneity and micro-heterogeneity. The former refers to discrete cell sub-populations, while the latter can be viewed as a spread in the distribution of the expression of a trait in an seemingly homogeneous cell population^6^. This kind of cell-to-cell variability has been reported to modulate some important physiological mechanisms as, for instance, the differentiation decisions of a multipotent mouse hematopoietic cell line and the asymmetric DNA segregation during muscle satellite cells (MuSCs) division^7,8^. However, the involvement of micro-heterogeneity in disease is only poorly explored.

We asked whether the heterogeneity observed in muscle interstitial cells has any impact on and is affected by myopathies such as Duchenne muscular dystrophy (DMD). DMD is an X-linked genetic disease caused by mutations in the dystrophin gene^9^. Sarcolemma instability originating from this condition leads to a chronic damage, progressive muscle wasting and deposition of ectopic tissues, such as intramuscular adipose tissue (IMAT) and fibrotic tissue^10,11^. The source of ectopic tissues is a population of interstitial cells known as fibro/adipogenic progenitors (FAPs)^12^ that develop from embryonic mesenchymal cells^13^. FAPs reside in the endomysium of skeletal muscle and are characterised by the expression of the surface proteins stem cell antigen-1 (SCA-1 also known as lymphocyte antigen 6A-2/6E-1, Ly6a) and CD140a (also known as platelet-derived growth factor receptor alpha, PDGFR-alpha)^14–17^. Recently, single-cell approaches have revealed heterogeneity in FAPs and have provided some evidence of its involvement in muscle development and dystrophy in mouse models^18–20^.

Here, we characterise two dynamic cell states in FAPs from muscles of *mdx* mice and describe how this heterogeneity impacts their properties ex vivo and in vivo. We show that the two FAP cell states, characterised by different levels of SCA-1 expression, differ in differentiation and proliferation potential. Furthermore, we show that the muscle microenvironment differentially modulates the behaviour of the two FAP cell states by affecting their propensity to differentiate into adipocytes in an age-dependent manner. These results suggest that micro-heterogeneity takes part in the fate decision of a mesenchymal cell population involved in the pathogenesis of a genetic disease.

## Materials and methods

### **M**ouse strains

C57BL/6J (wild type) and C57BL10ScSn-Dmd^mdx^/J (*mdx*) mice were purchased from the Jackson Laboratories (Ben Harbor, Maine, United States). We employed young (1.5 months old) and old (15 months old) mice. Whenever possible, we balanced gender of the mice in each experiment. Animals were bred and maintained according to the standard procedures of the animal facility. The experimental procedures were conducted according to the rules of good animal experimentation I.A.C.U.C. no. 432 of March 12 2006 and under the approval released from the Italian Ministry of Health.

### **I**solation of primary cells

Wild type and *mdx* mice were sacrificed through cervical dislocation and were washed with 70% ethanol. After an incision through the skin, hind limbs were excised and placed into cold Hank’s Balanced Salt Solution without Calcium and Magnesium (HBSS, Biowest, Nuaillé, France L0605-500) supplemented with 0.2% bovine serum albumin (BSA, Applichem PanReac, Darmstadt, Germany A1391) and 100 U/ml penicillin, 100 mg/ml streptomycin (Gibco, Waltham, Massachusetts, United States 15140122). Hind limb muscles were removed from bones under sterile hood and mechanically minced using a scalpel. Minced tissue was washed with HBSS and centrifuged at 700 × *g* for 10 min at 4 °C. Pelleted tissue was weighted and resuspended in the enzymatic digestion mix composed by 2,4 U/ml dispase II (4 ml/g of muscles) (Roche, Basel, Switzerland 04942078001) dissolved in Dulbecco’s phosphate buffered saline (D-PBS) with Calcium and Magnesium (Biowest L0625-500), 0,01 mg/ml DNase I (Roche 04716728001) and 2 μg/ml collagenase A (Roche 10103586001). Tissue preparations were incubated at 37 °C in a water bath (in gently shaking and not in immersion) for 1 h vortexing every 30 min. Digestion was stopped by the addition of HBSS and samples were centrifuged at 700 × *g* for 10 min at 4 °C. Pellets of cells were resuspended in 10 ml of HBSS and filtered through a 100 μm cell strainer (Falcon, New York, United States 352360). Cell suspensions were centrifuged at 700 × *g* for 10 min at 4 °C, pellets were resuspended in 10 ml of HBSS and filtered through a 70 μm cell strainer (Falcon 352350). After a further centrifuge red blood cells were lysed in 1 ml of RBC lysis buffer (Santa Cruz Biotechnology, Dallas, Texas, United States sc-296258) in ice for 2.5 min. Lysis was stopped adding HBSS and cell suspensions were filtered through a 40 μm cell strainer (Falcon 352340). Cell strainers were always washed before and after the use with 5 ml of HBSS. Cell suspensions were centrifuged at 700 × *g* for 10 min at 4 °C and resuspended in 500 μl of Magnetic Beads Buffer (MBB) composed by D-PBS without Calcium and Magnesium, 0.5% BSA and 2 mM Ethylenediaminetetraacetic acid (EDTA). Cells were filtered through a 30 μm cell strainer (Miltenyi, Bergisch Gladbach, Germany 130-041-407) which was washed 3 times with MBB. Mononuclear cells in this suspension were counted and centrifuged at 700 × *g* for 10 min at 4 °C. The isolation protocol proceeds with the magnetic activated cell sorting (MACS) of the CD45^−^ CD31^−^ cells. Pellets were resuspended in MBB and incubated with a microbead conjugated antibody against CD45 (Miltenyi 130-052-301) according to the manufacturer’s instructions. After 15 min, cells were washed with 2 ml of MBB and centrifuged. Pellets were resuspended in 500 μl of MBB and cells were separated with MS columns (Miltenyi 130-042-201) according to the manufacturer’s instructions to collect CD45^−^ cells. Protocol proceeds with the selection of CD31^−^ cells applying the same procedures described for CD45^−^ cells. Next, FAPs and muscle satellite cells (MuSCs) were isolated through fluorescent activated cell sorting (FACS). CD45^−^ CD31^−^ cell suspensions were incubated with the following primary antibodies for 30 min at 4 °C in D-PBS without Calcium and Magnesium, 2% BSA and 2 mM EDTA at 1 × 10^6^ cells per 100 μl: anti-ITGA7 APC 1:500 (Invitrogen, Waltham, Massachusetts, United States MA5-23555) and anti-SCA-1 FITC 1:50 (BD Pharmingen, Franklin Lakes, New Jersey, United States 557405). Gates were strictly designed using proper fluorescence minus one (FMO) controls for each antibody. Debris and cell clusters were excluded using side scatter (SSC) and forward scatter (FSC) as shown in supplementary figures. Moreover, we applied two criteria to enhance the reproducibility of the sorting strategy of FAP cell states: FAP cell states must represent at least 80% of the total FAPs (these criteria also increase the yield of the isolation); the mean intensity of SCA-1 must differ of at least 3-fold between the two cell states. FAPs and MuSCs were sorted directly in their growth media as ITGA7^−^ SCA-1^+^ and ITGA7^+^ cells respectively. The purity of sorted cells was assessed immediately after sorting.

Sorting was performed on FACSAria^TM^ III (Becton Dickinson) cell sorter equipped with 5 lasers (18 parameters). Analysis was carried out using FlowJo^TM^ Software.

### **F**AP cell states in vivo transplantation

A total of 75,000 freshly isolated SCA1-High-FAPs and SCA1-Low-FAPs were separately stained by incubating in the cell proliferation dye eFluor 670 (eBioscience 65-0840-85) following the manufacture’s instruction. SCA1-High-FAPs and SCA1-Low-FAPs were then resuspended in 30 μl of PBS and injected into the left and right TAs of *mdx* mice, respectively. After three days, the TAs and a gastrocnemius were harvested and total mononuclear cells were isolated as already described. Cells were then incubated 30 min in ice with the anti-SCA-1 FITC antibody and analysed by flow cytometry. Gastrocnemius was used to determine the gate identifying labelled cells.

### **E**dU administration

5-ethynyl-2′-deoxyuridine (EdU) (Invitrogen, C10418) was dissolved with 2 ml sterile PBS at the concentration of 5 mg/ml (20 mM). Mice were weighted and injected intraperitoneally with 40 mg/kg EdU^21^. After 24 h, CD45^−^ CD31^−^ were isolated by MACS and staining was performed using Click-iT^TM^ EdU Pacific Blue^TM^ Flow Cytometry Assay Kit (Invitrogen, C10418). Briefly, 1 × 10^6^ CD45^−^ CD31^−^ were incubated with anti-ITGA7 APC 1:500 (Invitrogen MA5-23555) and anti-SCA-1 FITC 1:50 (BD Pharmingen 557405) antibodies for 30 min in ice. The subsequent fixation, permeabilization and Click-iT^TM^ reaction were performed as indicated by the manufacturer’s instructions. Unstained cells and FMO controls were prepared. Samples were resuspended at the concentration of 1 × 10^6^ cells per ml in PBS 1% BSA and analysed as described in the flow cytometry section.

### **F**low cytometry

Cell suspensions were incubated with primary antibodies in ice for 30 min in PBS containing 2 mM EDTA and 2% BSA at the concentration of 1 × 10^6^ cells/ml. We used the following antibodies and dilutions: anti-ITGA7 APC 1:500 (Invitrogen MA5-23555), anti-SCA-1 FITC 1:50 (BD Pharmingen 557405) and anti-CD140a APC 1:50 (eBioscience, San Diego, California, United States 17-1401). To stop the incubation cell suspensions were diluted with 2 ml of PBS 2 mM EDTA 2% BSA and centrifuged at 4 °C for 10 min at 700 × *g*. Pellets were resuspended in PBS 2 mM EDTA 2% BSA at the concentration of 1 × 10^6^ cells/ml. Unstained samples and suitable FMO controls were prepared when necessary. Approximately 10,000 events per samples were acquired by CytoFLEX S (Beckman Coulter, Brea, California, United States) equipped with three lasers (488 nm, 405 nm and 638 nm) and 13 detectors. Quality control of the cytometer was assessed daily using CytoFLEX Daily QC Fluorospheres (Beckman Coulter B53230). Data were collected by CytExpert (Beckman Coulter) software. If needed, a compensation matrix was calculated using VersaComp Antibody Capture Kit (Beckman Coulter B22804) according to the manufacturer’s instructions. FCS files were analysed using FlowJo^TM^ Software.

### **M**ultiplex flow cytometry assay and analysis

Total mononuclear cells were isolated from wild type and *mdx* mice as described. 1 × 10^6^ cells were stained in PBS containing 2 mM EDTA and 2% BSA at the concentration of 1 × 10^6^ cells/ml in ice for 30 min with antibodies reported in Supplementary Table 1. 2 ml of PBS 2 mM EDTA 2% BSA were added to stop the incubation, cell suspensions were centrifuged and cell pellets were suspended in PBS containing 2 mM EDTA and 2% BSA. At least 300,000 events were acquired by CytoFLEX S (Beckman Coulter) and collected by CytExpert (Beckman Coulter) software. Wild type and *mdx* mice samples were acquired at different days therefore we created two different compensation matrices. Next FCS files of these two datasets were simultaneously uploaded on Cytobank platform to perform multidimensional analysis. Cells were identified by SSC-A and FCS-A and single cells by FCS-A and FSC-H applying ‘tailored’ gates for each sample.

Dimensional reduction was carried out applying the tSNE^22^ algorithm as followed: 50,000 events per sample; 2000 iterations; perplexity = 30; theta = 0.5. All 9 channels were selected applying compensation matrices.

To identify main muscle populations firstly we ran the FlowSOM algorithm with the following settings: all markers were selected; 50,000 events per sample; 100 clusters; 25 metaclusters; 10 iterations with scale normalisation. A hierarchical consensus clustering method was used to obtain the metaclusters. Next, metaclusters with similar expression profiles were merged together using the ‘automated cluster gates’ function.

FAP heterogeneity was studied applying the FlowSOM algorithm as followed: 600 events per samples; selected markers were specified in the results section; 100 clusters; 4 metaclusters; 10 iterations with scale normalisation. A hierarchical clustering method was used to obtain the metaclusters. We merged the two metaclusters expressing higher levels of SCA-1 and the two metaclusters expressing lower levels of SCA-1 obtaining two groups of FAPs. Self-organising maps (SOM) were uploaded in Cytoscape^23^ and graph layouts were modified.

### **M**ass cytometry

Isolation of FAPs by MACS technology from *mdx* mice was performed according to the protocol described in Cerquone Perpetuini et al., 2020.

FAPs were incubated with Cell-ID-Cisplatin-194Pt (Fluidigm, South San Francisco, California, United States, 201194) at the concentration of 1 μM to stain dead cells. Next, samples from three independent biological replicates were barcoded with a combination of palladium isotopes of the Cell-ID 20-Plex Pd Barcoding kit (Fluidigm 201060) at the concentration of 5 μM. Staining was quenched by adding Maxpar cell staining buffer (Fluidigm 201068). Barcoded samples were mixed and stained with lanthanides-conjugated antibodies according to the manufacturer’s instructions. The full list of antibodies purchased from Fluidigm is reported in Supplementary Table 2. Next, the suspension was incubated with Cell-ID Intercalator-Ir (Fluidigm, 201192B) at the concentration of 125 nM for 1 h in Maxpar fix and Perm Buffer (Fluidigm 201067). Finally, cells were filtered with 30 μm cell strainer.

Samples were acquired using CyTOF2 tuned and calibrated according to the manufacturer’s instructions. Rate of acquisition was < 400 events per second. Dataset was converted in fcs files using Debarcoding software (Fluidigm) and normalised.

### **A**nalysis of mass cytometry data

Debarcoded fcs files were uploaded to Cytobank platform. Cells were identified using DNA1 and DNA2 content identified by Ir intercalator, while single cells were identified using the event length parameter and DNA1 (or DNA2). Next, dead cells were gated out using Pt channel.

For the experiment with FAPs isolated by MACS and induced to differentiate into adipocyte, dimensional reduction was carried out applying the tSNE algorithm as followed: 35,000 events per sample; 1000 iterations; perplexity = 30; theta = 0.5. All parameters were selected as clustering channels. To identify FAP cell states we ran the FlowSOM algorithm with the following settings: SCA-1 and phospho-CREB-1 were selected as clustering channels; all events were selected; 100 clusters; 3 metaclusters; 10 iterations. A k-Means clustering method was used to obtain the metaclusters. Next, metaclusters with similar expression profiles were merged together using the ‘automated cluster gates’ function and mapped onto viSNE maps to facilitate results interpretation.

### **C**ell culture

Freshly isolated *mdx* FAPs cell states were seeded on 96 well plates (Falcon 353072) in growth medium (GM) at the density of 15,000 cell/cm^2^ for differentiation assays and at the density of 6200 cells/cm^2^ for the proliferation assay. GM is composed by Dulbecco’s Modified Eagle Medium, High Glucose, GlutaMAX^TM^ (Gibco 61965-026) supplemented with 20% fetal bovine serum (FBS) (Euroclone, Pero, Milan, Italy, ECS0180L), 100 U/ml penicillin and 100 mg/ml streptomycin (Gibco 15140122), 1 mM sodium pyruvate (Sigma-Aldrich, St. Louis, Missouri, United States, S8636) and 10 mM 4-(2-hydroxyethyl)-1-piperazineethanesulfonic acid (HEPES) (Sigma H0887).

To induce adipogenic differentiation cells were exposed to the adipogenic induction medium (AIM) consisting of GM complemented with 1 μg/ml insulin (Sigma-Aldrich I9278), 1 μM dexamethasone (Sigma D4902) and 0.5 mM of 3-isobutyl-1-methylxanthine (IBMX) (Sigma, I5879). After three days, cells were exposed to the maintenance medium (MM) composed by GM and 1 μg/ml insulin for additional two days. For the adiponectin experiment, the two FAP cell states were treated with 25 μM AdipoRon (Selleckchem, Munich, Germany S7365) for 24 h in GM before adipogenic induction.

To induce fibrogenic differentiation cells were cultured in a medium composed by Dulbecco’s Modified Eagle Medium, High Glucose, GlutaMAX^TM^, 100 U/ml penicillin and 100 mg/ml streptomycin, 1 mM sodium pyruvate and 10 mM HEPES, 5% horse serum (HS) (Euroclone ECS0090D) and 1 ng/ml TGF-beta-1 (PreproTech, Cranbury, New Jersey, United States 100-21). To study the expression of collagen genes, cells were plated in 24 well plates at the concentration of 15,000 cells/cm^2^.

For in vitro expansion, FAPs cell states were cultured at the concentration of 3400 cells/cm^2^ for 9 days in Cytogrow (Resnova, Genzano di Roma, Italy TGM-9001-B) medium. Afterwards, cell monolayers were washed twice in sterile D-PBS and cells were detached with Trypsin 0.5 g/l EDTA 0.2 g/l (Lonza, Basel, Switzerland # BE17-161E) for 5 min. Next, cells were processed for flow cytometry analysis.

Muscle satellite cells (MuSCs) were cultured in a growth medium composed by Dulbecco’s Modified Eagle Medium, High Glucose, GlutaMAX^TM^, 100 U/ml penicillin and 100 mg/ml streptomycin, 1 mM sodium pyruvate, 10 mM HEPES, 20% FBS, 10% Horse serum (Euroclone ECS0090D), 2% Chicken embryo extract (Biotrend, Koln, Germany CE-650-J).

CD45^+^ leucocytes were isolated from *mdx* mice with MACS technology. They were cultured for 24 h in DMEM Dulbecco’s Modified Eagle Medium, High Glucose, GlutaMAX^TM^, 100 U/ml penicillin and 100 mg/ml streptomycin with 0.2% BSA^24^ at 375,000 cells/ml. Next, media were harvested, centrifuged at 700 × *g* for 10 min at 4 °C and finally supernatant was filtered to remove cell debris. Conditioned media from different biological replicates were mixed just before the treatment. One volume of the resulting conditioned medium was mixed with one volume of 2X AIM to induce adipogenesis. The same procedure was applied to prepare MM.

### **I**mmunofluorescence

Indirect immunolabelling was performed as followed. Cells were fixed with 2% paraformaldehyde (Santa Cruz sc-281692) for 20 min at room temperature (RT). Paraformaldehyde was removed by inversion and cells were washed three times in PBS and one time in PBS 0.5% Triton X-100. Permeabilization was performed incubating cells for 5 min with 0.5% Triton X-100 (Sigma T9284) in PBS. Permeabilization solution was removed by inversion and cells were washed with 0.1% Triton X-100 in PBS. Unspecific sticky sites were blocked with a blocking solution consisting of 10% FBS 0.1% Triton X-100 in PBS for 30 min at RT. Next, samples were incubated with the following primary antibodies for 1 h at RT: rabbit anti-PPAR-gamma 1:200 (Cell Signaling. Danvers, Massachusetts, United States 2443 S), mouse anti-SMA 1:300 (Sigma A5228) and rabbit Ki-67 1:400 (Cell Signaling 9129). For CD140a immunolabelling, cells were incubated overnight at 4 °C with the anti-CD140a (R&D Systems, Minneapolis, Minnesota, United States AF1062) diluted 1:80 in blocking solution. Then samples were washed three times with 0.1% Triton X-100 in PBS for 5 min and were incubated 30 min at RT with the following secondary antibody diluted 1:200: goat anti-rabbit Alexa Fluor 488 (Southern Biotech, Birmingham, Alabama, United States 4050-30), goat anti-mouse Alexa Fluor 488 (Southern Biotech 1030-30) donkey anti-goat Alexa Fluor 488 (Southern Biotech 3425-30). Samples were washed three times with PBS 0.1% Triton X-100 for 5 min and nuclei were counterstained with 2 μg/μl Hoechst 33342 (Invitrogen H3570) in PBS 0.1% Triton X-100 for 5 min at RT. Finally, cells were washed three times in PBS for 5 min and samples were stored at 4 °C in PBS containing 0.02% of sodium azide.

For immunolabelling on muscle section, tibialis anteriors (TAs) were embedded in optical cutting temperature (OCT) compound (Bio-optica, Milan, Italy 05-9801) and snap frozen in liquid nitrogen. Embedded tissues were stored at −80 °C. TAs were sectioned using a cryostat (Leica, Wetzlar, Germany) to produce transverse sections of 8–10 μm thickness. Sections were stored at −80 °C. Sections were fixed in 4% PFA for 10 min at RT and washed three times with PBS for 5 min. Next, permeabilization was performed incubating sections with 0.3% Triton X-100 in PBS for 30 min at RT. Unspecific sticky sites were blocked by a blocking solution composed of 0.1% Triton X-100 1% glycine (SERVA, Heidelberg, Germany 23390.02) 10% goat serum (Euroclone ECS0200D) in PBS for 1 h at RT. Anti-perilipin antibody (Cell signalling 3470) was diluted 1:100 in blocking solution and incubated with sections overnight at 4 °C. Sections were washed twice in 0.2% Triton X-100 1% BSA in PBS (washing solution) for 15 and 5 min at RT. Incubation with goat anti-rabbit Alexa Fluor 488 1:200 (Southern Biotech 4050-30) diluted in blocking solution was performed at RT for 30 min. At the end of incubation, sections were washed 15 min in washing solution and 5 min in PBS. Finally, nuclei were stained with 2 μg/μl Hoechst 33342 (Invitrogen H3570) in PBS for 10 min at RT. Sections were washed twice in PBS for 5 min. Finally, slides were covered with coverslips using Aqua-Poly/Mount (Tebu-bio 18606-20) and stored at 4 °C.

For the on-section detection of FAPs, TAs section were fixed in 2% PFA and washed three times with PBS. Permeabilization was performed incubating sections with 0.3% Triton X-100 in PBS for 30 min. This step was directly followed by an incubation with Protein Block Serum Free (Dako X0909) for 30 min to block the sticky sites. After a wash in PBS for 5 min, sections were incubated overnight at 4 °C with the following antibodies diluted in PBS 2.5% BSA: goat anti-CD140a 1:80 (R&D Systems AF1062) and rabbit anti-PPAR-gamma 1:200 (Cell Signaling 2443S). The following day section was washed three times for 10 min with PBS 0.1% Triton X-100. Next, samples were incubated for 30 min with the following secondary antibodies diluted in PBS 2.5% BSA: donkey anti-goat Alexa Fluor 488 1:200 (Southern Biotech 3425-30) and donkey anti-rabbit Alexa Fluor 555 1:200 (Thermo fisher scientific A32794). After two wash for 5 min each with PBS 0.1% Triton X-100 and three wash in PBS, sections were incubated with 2 μg/μl Hoechst 33342 (Invitrogen H3570) in PBS for 10 min at RT. Sections were washed three times in PBS for 5 min. Finally, slides were covered with coverslips using Aqua-Poly/Mount (Tebu-bio, Le Perray-en-Yvelines, France 18606-20) and stored at 4 °C.

### **O**il red O staining

Oil red O (ORO) (Sigma-Aldrich O0625) was dissolved in isopropanol at the concentration of 3.5 mg/ml. This stock was diluted to a working solution composed of 3 volumes of ORO and 2 volumes of distilled water. This ORO solution was filtered two times just before the use. Fixed cells were washed three times in PBS for 5 min and were incubated with ORO for 10 min at RT. ORO was removed by inversion and cells were washed three times in PBS for 5 min. Nuclei were counterstained with 2 μg/μl Hoechst 33342 in PBS 0.1% Triton X-100 for 5 min at RT. Finally, cells were washed three times in PBS for 5 min and were stored at 4 °C in PBS containing 0.02% of sodium azide.

During immunofluorescence, ORO staining was performed after incubation with secondary antibodies. After three washes in PBS protocol continues with the Hoechst 33342 incubation as described in the immunofluorescence section.

### **A**lkaline phosphatase assay

The two FAP cell states were isolated from *mdx* mice and cultured for 5 days in GM at 15,000 cell/cm^2^. Samples were fixed using 2% paraformaldehyde and then washed three times with PBS. NBT/BCIP stock solution (Roche 11681451001) was diluted 1:50 in a buffer composed of 0.1 M Tris, 0.05 M MgCl_2_, 0.1 M NaCl (pH = 9.5) and added to cell monolayers. After 10 min this buffer was removed and samples were washed three times in PBS. Finally, nuclei were stained with Hoechst 33342 as already described.

### **C**FSE staining and analysis of cell morphology

CarboxyFluorescein succinimidyl ester (CFSE) (Abcam, Cambridge, United Kingdom ab113853) staining was carried out according to the manufacturer’s instructions. Culture medium was removed and then 10 μM of CFSE in PBS was overlaid onto cells. After 15 min of incubation at 37 °C, staining was quenched adding an equal volume of GM and allow to sit for 5 min at 37 °C. Next medium was removed and cells were washed one time in PBS and fixed with 2% paraformaldehyde. Samples were washed three times in PBS for 5 min. Nuclei were counterstained with 2 μg/μl Hoechst 33342 in PBS 0.1% Triton X-100 for 5 min at RT. After three wash in PBS samples were at 4 °C in PBS containing 0.02% of sodium azide.

Images were manually acquired. Images were analysed using Fiji. Background signal was removed using the subtract background command and an image specific threshold was applied to obtain a binary image. Then we run the following series of commands to define cell borders: median filter, erode and fill holes. Finally, we measured area occupied, roundness and aspect ratio. We analysed at least 50 cells^25^ from a total of three independent biological replicates.

### **A**cquisition of images and analysis

Images were acquired with a LEICA microscope (DMI6000B) using the LAS X software. For experiments using ex vivo cell culture, a matrix of 25 non-overlapping field per well was acquired. Experiments were performed in technical duplicate. ORO positive cells, Ki-67 positive nuclei and PPAR-gamma positive nuclei were manually counted using Fiji and were divided by the number of nuclei per field. SMA-positive area per cell was assessed using a dedicated pipeline of CellProfiler^26^. Area expressed in pixel was converted in square micron and normalised over the total number of nuclei in each field.

Microphotographs of TA sections immunolabelled for perilipin were acquired using a 10x objective. At least three sections for each mouse were acquired. These overlapped images were used to reconstruct the whole TA section through the grid/collection stitching algorithm available in Fiji. To identify perilipin positive area we applied a threshold (0-100) and we divided this area by the whole section area.

Microphotographs of TA section labelled for CD140a were acquired with Leica microscope for the quantification and representative images were acquired using the confocal microscope Olympus IX-81 with a 40x objective.

Images of CD140a and PPAR-gamma positive FAPs on the TA section were acquired using the confocal microscope Olympus IX-81 with a 40x objective.

### **R**NA extraction, retrotranscription and Real-Time PCR

Cell monolayers were washed in PBS and lysed in TRIzol^TM^ Reagent (Invitrogen 15596-018). For extraction from tissue, TAs were homogenised in liquid nitrogen using a pestle and then resuspended in TRIzol^TM^. Isolation of total RNA was performed according to the manufacturer’s instruction. RNA precipitation was performed at −20 °C overnight with glycogen. Total RNA was resuspended in nuclease free water, concentration and 260 nm/280 nm ratio were determined by Nanodrop Lite Spectrophotometer (Thermo Fisher Scientific, Waltham, Massachusetts, United States). Samples were diluted to the concentration of 50 ng/μl and stored at −80 °C. cDNA was generated by PrimeScript RT Reagent Kit (Takara, Kusatsu, Japan RR037A). Real-time PCR was performed on 15 ng of cDNA using SYBR Premix Ex Taq (Tli RNaseH Plus) (Takara RR420W) in a reaction volume of 20 μl. Tubulin was used as housekeeping gene. We used primers in Supplementary Table 3. Comparative expression was performed using the 2^-ΔΔCt^ method^27^ where ΔΔ*C*_t_ is (*C*_t, target_ − *C*_t, tubulin_) − (*C*_t, mean target_ − *C*_t, mean tubulin_).

### **R**NA-sequencing experiment

FAP cell states were cultured 5 days in GM at the density of 15,000 cell/cm^2^. Next, media were removed, cell monolayers were washed twice in PBS and detached using Trypsin 0.5 g/l EDTA 0.2 g/l. Total RNA was isolated from the harvested cells using RNeasy micro kit (Qiagen, Hilden, Germany 74004) and was quantified using the Qubit 2.0 fluorimetric Assay (Thermo Fisher Scientific). Libraries were prepared from 100 ng of total RNA using the QuantSeq 3′ mRNA-Seq Library Prep Kit FWD for Illumina (Lexogen GmbH, Vienna, Austria) and their qualities were assessed by using screen tape High sentisivity DNA D1000 (Agilent Technologies, Santa Clara, California, United States). Libraries were sequenced on a NovaSeq 6000 sequencing system using an S1, 100 cycles flow cell (Illumina Inc., San Diego, California, United States). Illumina novaSeq base call (BCL) files were converted into fastq file by bcl2fastq (version v2.20.0.422). Sequence reads were trimmed using bbduk software (bbmap suite 37.31) in order to remove adapter sequences, poly-A tails and low-quality end bases (regions with average quality below 6). Alignment was performed with STAR 2.6.0a^28^ on mm10 reference assembly obtained from cellRanger website (Ensembl assembly release 93). The expression levels of genes were determined with htseq-count 0.9.15 (Ref. ^29^) by using mm10 Ensembl assembly (release 93) downloaded from cellRanger website. The expression data was analysed by Rosalind, with a HyperScale architecture developed by OnRamp BioInformatics. Individual sample counts were normalised via Relative Log Expression (RLE) using DESeq2 R library^30^. Multidimensional scaling (MDS) plot was generated as part of the QC step. DEseq2 was also used to calculate fold changes and *p*-values and perform covariate correction. Clustering of genes for the final heatmap of differentially expressed genes was done using the PAM (Partitioning Around Medoids) method using the fpc R library. Gene set enrichment analysis was performed using DAVID^31^. PPAR-gamma transcriptionally regulated genes have been retrieved from TRRUST^32^. Cystoscape has been applied to create the networks.

The RNA-seq data have been deposited in GEO with the identifier GSE163016.

### **S**ingle-cell RNAseq data analysis

Briefly, single-cell RNA-seq data of hind limb muscle have been retrieved from Tabula Muris Senis project and re-processed with the Seurat R package^33^. After log normalisation, we used the 2000 most variable features to proceed to downstream analyses. Data have been scaled and the first 10 principal components used to run the t-distributed stochastic neighbour embedding (tSNE) algorithm. Whenever possible, biomarkers have been retrieved from Myo-REG database to assign cell line ontology identifications to each cluster, on the basis of the expression level of each biomarker for each cluster, according to their distribution. Once cell IDs have been assigned, FAPs population subset has been further analysed for the expression of SCA-1 and downstream analyses.

Differentially expressed genes were identified by comparing the mRNA expression profile of the two FAPs clusters. Only genes whose expression difference had an adjusted *p*-value < 0.05 were considered as sub-population biomarkers. EnrichR^34^ has been used to retrieve the enriched GO Molecular Function terms for each population with *p*-value < 0.05.

### **S**tatistical analysis

Experiments were performed with primary cells isolated from at least three different mice (here defined biological replicates). The number of biological replicates was specified in the figure legends. No statistical methods were applied to estimate sample size and the investigator was not blinded to the group of allocation during the experiments. Statistical analysis was performed by a two-tailed Student’s *t* test between two groups, while one-way ANOVA or two-way ANOVA were applied for comparison among more than three groups. Results were presented as mean ± standard error of the mean (SEM). Differences were considered significant when *p*-value is less than 0.05 (*p ≤ 0.05, **p ≤ 0.01, ***p ≤ 0.001, ****p ≤ 0.0001). Plots and statistical analysis were produced using the GraphPad Prism 7 software.

## Results

### **S**CA-1 expression defines two FAP populations

**Fig. 1:**
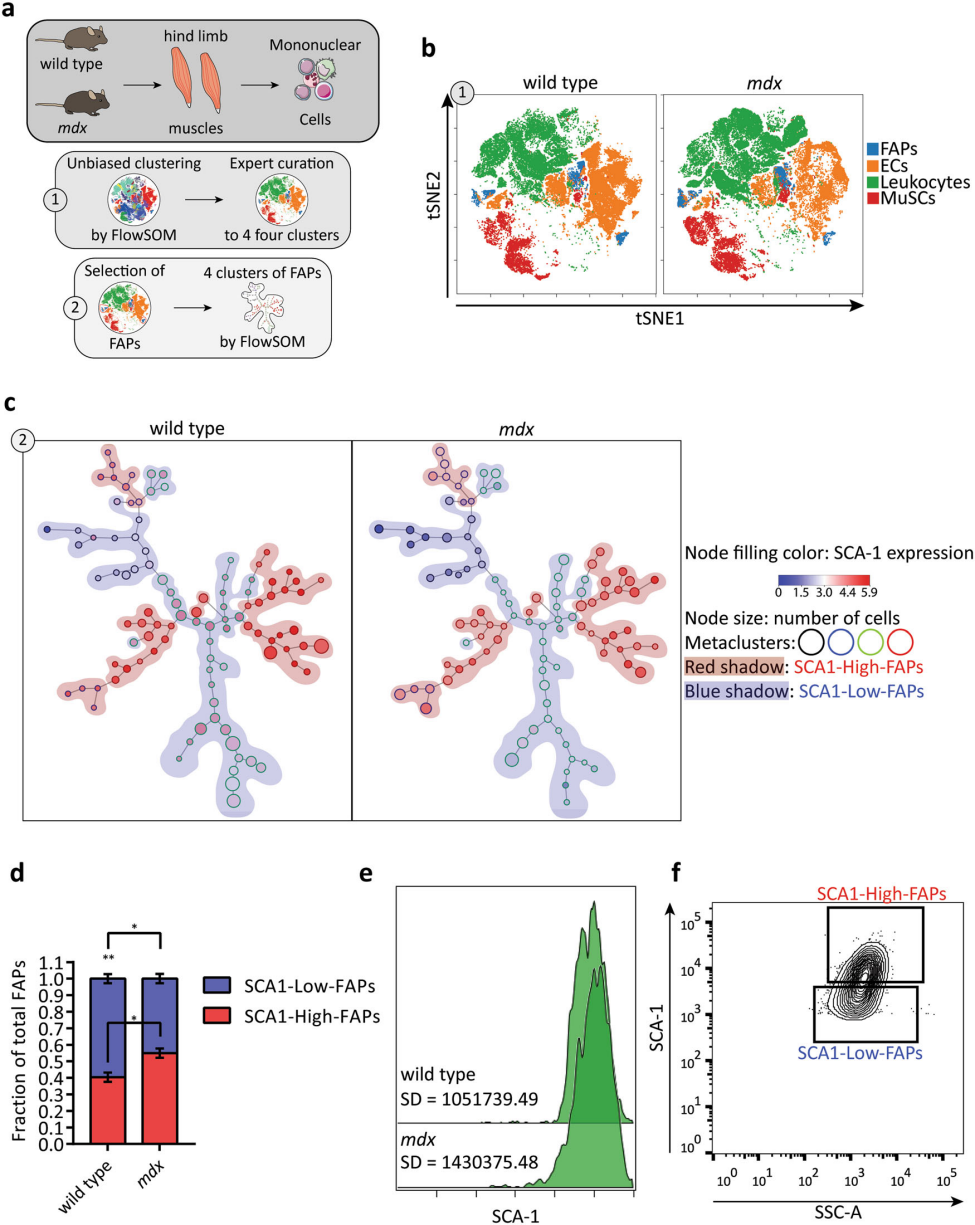
Micro-heterogeneity of SCA-1 in fibro/adipogenic progenitors. **a** Schematic representation of the workflow applied to study FAP heterogeneity. Cells are taken from Servier Medical Art (SMART), under Creative Commons Attribution 3.0 Unported License. **b** Representative viSNE maps of total mononuclear cells isolated from young wild type and *mdx* mice. The four clusters produce by FlowSOM algorithm were mapped onto the viSNE maps and correspond to the following mononuclear cell populations: FAPs (blue), endothelial cells (orange), leucocytes (green) and muscle satellite cells (MuSCs, red). **c** Representative Self-Organising Maps (SOMs) of FAPs identified in **(a)**. SOMs were obtained with the FlowSOM algorithm. Each node represents a cluster of cells, nodes with similar expression profile are linked by an edge. Colour of nodes indicate SCA-1 expression level. Node outlines indicate the four metaclusters (red, blue, black and green) obtained by the algorithm. Red and blue shadings highlight FAPs expressing high levels (red and blue metaclusters, called SCA1-High-FAPs) and low levels (black and green metaclusters, called SCA1-Low-FAPs) of SCA-1. **d** Stacked bar plot showing the fraction of SCA1-High-FAPs and SCA1-Low-FAPs in wild type and *mdx* mice. Data are presented as mean ± SEM. Statistical significance was estimated by a One-way ANOVA, *p ≤ 0.05, **p ≤ 0.01 (*n* = 4). **e** Representative SCA-1 histograms of FAPs from wild type and *mdx* mice identified in (**b**) and their standard deviations (SD) showing a typical micro-heterogeneity profile. **f** Sorting strategy to decompose SCA-1 micro-heterogeneity and to isolate SCA1-High-FAPs and SCA1-Low-FAPs from *mdx* mice. Complete strategy, Fluorescence minus one (FMO) controls and cell states purity in Supplementary Fig. 3.

We have recently shown that purified FAPs are a heterogeneous population including several sub-populations^19^. Here we use a multiplex flow cytometry approach to characterise FAP heterogeneity from a suspension of mixed populations derived from skeletal muscles. We first isolated mononuclear cells from hind limb muscles of 6-8 weeks old wild type and *mdx* mice. In this time window, muscles of dystrophic mice are in a regeneration phase^35,36^. Cells were stained with fluorescent antibodies against 9 surface antigens (CD45, CD31, Integrin alpha-7, SCA-1, CD140a, CD140b, CD146, CD34, CD90.2) to distinguish the main mononuclear cell populations residing in the skeletal muscle: leucocytes (CD45^+^), endothelial cells (CD45^−^ CD31^+^), muscle satellite cells (MuSCs) (CD45^−^ CD31^−^ Integrin alpha-7^+^) and FAPs (CD45^−^ CD31^−^ Integrin alpha-7^−^ SCA-1^+^). The antigen expression profiles of single cells were analysed by a mixed approach whereby an automatic clustering is followed by expert manual refinement (Fig. 1a and details in supplementary materials). This approach yielded 4 cell clusters corresponding to the main muscle mononuclear cell populations (Fig. 1b) and 11 unassigned clusters (Supplementary Fig. 1d), which were characterised by an antigen profile that could not be assigned to any of the main mononuclear cell populations in the muscle niche. FAPs amount to 2.6% of the mononuclear cells in the wild type mouse, whereas their abundance increases to 5.7% in the *mdx* mouse (Supplementary Fig. 1e).

We next focused on FAPs and we chose to study the expression variability of 6 antigens (SCA-1, CD140a, CD140b, CD146, CD34, CD90.2) associated to the FAP immunophenotype^14,15,19,20^. By applying the same clustering strategy used for the total mononuclear populations, we obtained 4 metaclusters represented by red, blue, green and black outlined nodes of the self-organising maps (SOMs) produced by the FlowSOM algorithm (Fig. 1c and Supplementary Fig. 2). Interestingly, we noticed metaclusters that express high level of SCA-1 (red and blue outlined nodes highlighted by red shading in Fig. 1c) and metaclusters expressing low level of SCA-1 (black and green outlined nodes highlighted by blue shading in Fig. 1c). These two FAP populations are equally represented in the *mdx* mouse, while FAPs expressing low level of SCA-1 are predominant in the wild type model (Fig. 1d). The intensity distribution of SCA-1 shows a single peak and a large standard deviation (Fig. 1e), features that characterise the cell to cell variability dubbed micro-heterogeneity^6^.

### **S**CA1-High-FAPs and SCA1-Low-FAPs are cell states in dynamic equilibrium

The observation that FAPs display micro-heterogeneity prompted us to design a sorting strategy to isolate, from the *mdx* mouse model, FAPs (CD45^−^ CD31^−^ Integrin alpha-7^−^ SCA-1^+^) expressing high and low levels of SCA-1, henceforth called SCA1-High-FAPs and SCA1-Low-FAPs respectively (Fig. 1f; refer to Supplementary Fig. 3a for the complete gating strategy, to the Supplementary Fig. 3b, c for controls and population purity). Similar sorting strategies have already been used to resolve micro-heterogeneity^7,8,37^. Sorted FAP populations are significantly enriched for CD140a, as assessed by immunofluorescence (Supplementary fig. 3d) and by flow cytometry (Supplementary Fig. 3e, f). Both populations can be stained for alkaline phosphatase (Supplementary Fig. 3g) but they do not show any myogenic potential (Supplementary Fig. 3h).

Next, we asked whether FAPs expressing high and low levels of SCA-1 represent stable sub-populations or rather dynamic cell states. Terminology in the cell plasticity field is still debated^38^. Here we define a “sub-population” of a given cell type as a group of cells identified by a discrete expression of specific markers and capable of maintaining identity when grown for several duplications ex vivo. Instead, considered a seemingly homogeneous cell population, we talk of a “cell state” when referring to a group of cells with an expression profile skewed when compared to the symmetrical expression distribution of the parent population. Cell states are in a dynamic equilibrium therefore they lose their identity after in vitro expansion^7^.

**Fig. 2:**
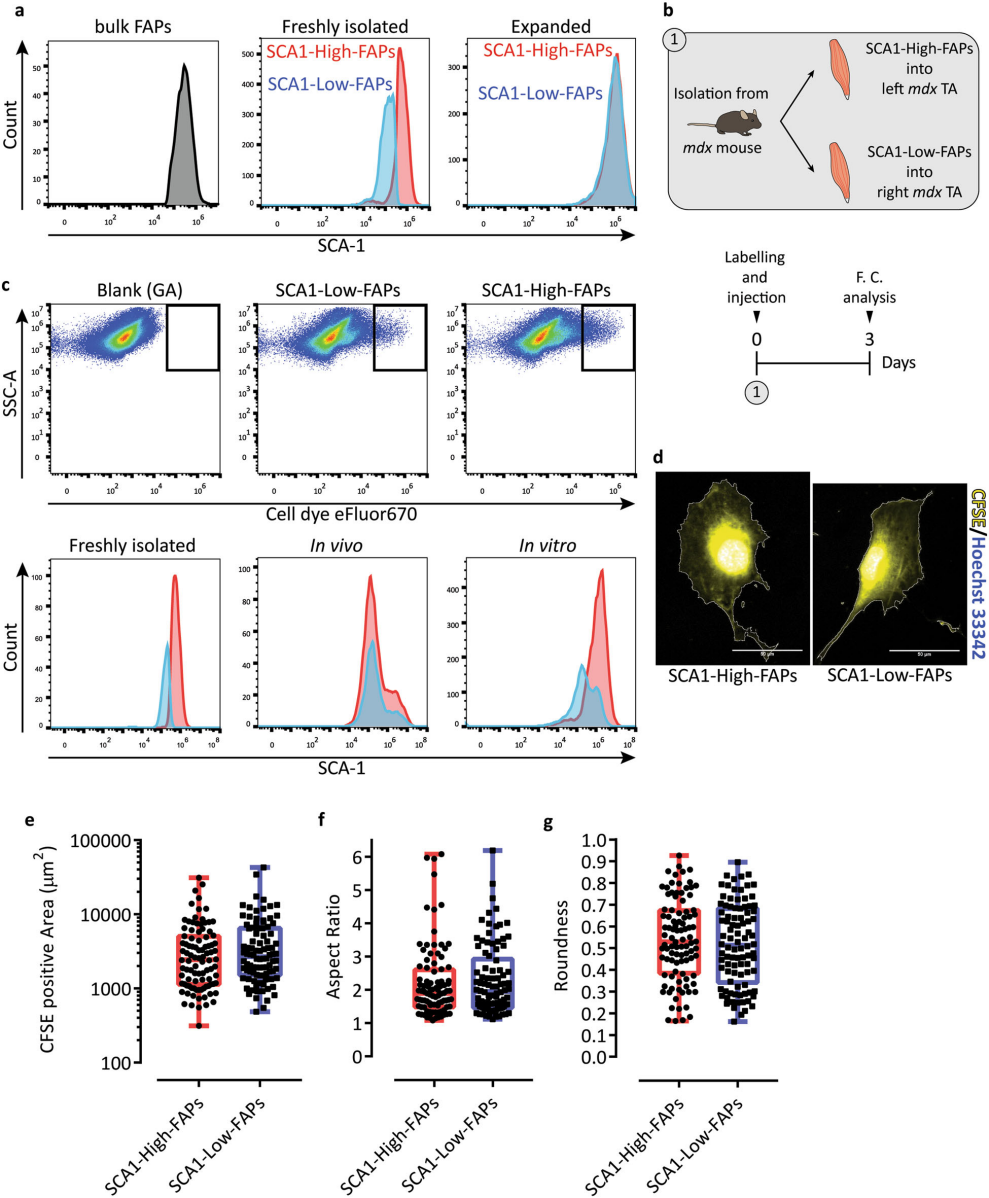
SCA1-High-FAPs and SCA1-Low-FAPs from *mdx* mouse are two cell states. **a** SCA-1 histogram of the whole FAP compartment (left), freshly isolated cell states (middle) and cell states expanded 9 days in Cytogrow (right). FAP compartment was analysed as followed: CD45^−^ CD31^−^ cells isolated by MACS were stained with antibodies against Integrin alpha-7 and SCA-1; next a gating strategy (Supplementary Fig. 4b; unstained cells in Supplementary Fig. 4a) was applied to obtain Integrin alpha-7^−^ SCA-1^+^ cells. Cell states were isolated as described and then analysed by flow cytometry (gating strategy in Supplementary Fig. 4d, e for SCA1-High-FAPs and SCA1-Low-FAPs respectively; controls in Supplementary Fig. 4c) (*n* = 3). **b** Schematic representation of the in vivo experiment. Freshly isolated FAP cell states were stained and injected into TAs of *mdx* mice. After 3 days SCA-1 expression of stained cells was studied by flow cytometry. **c** Upper panel: dot plots showing stained FAP cell states three days after the injection. Lower panel: SCA-1 distributions of FAP cell states in three different condition: freshly isolated, after 3 days in vitro and after 3 days in vivo (*n* = 3). **d** Representative crop of micrographs of SCA1-High-FAPs (left) and SCA1-Low-FAPs (right) cultured ex vivo. Cytoplasms were stained with CFSE (yellow) and nuclei were counterstained with Hoechst 33342 (blue). Scale bar 50 μm. **e**–**g** Box plots representing CFSE positive area, aspect ratio (AR) and roundness of SCA1-High-FAPs (n = 96) and SCA1-Low-FAPs (n = 94) from three different biological replicates. Whiskers are minimum and maximum values of the distribution. Statistical significance was estimated by a two tailed Mann–Whitney test after a normality test.

To address this issue, we performed a repopulation experiment in which SCA1-High-FAPs and SCA1-Low-FAPs were allowed to proliferate ex vivo. After 9 days, we analysed SCA-1 expression by flow cytometry. The SCA-1 intensity distributions of freshly isolated cells are clearly separated, whereas in expanded cells they overlap and restore the expression profile of the whole FAP compartment (Fig. 2a, gating strategies and controls in Supplementary Fig. 4a–e). We also explored the dynamics of the two populations in vivo by injecting purified and fluorescently labelled FAP populations into the tibialis anteriors (TAs) of *mdx* mice. Three days after transplantation, total mononuclear cells were isolated from TAs and the distribution of SCA-1 expression was evaluated by flow cytometry (Fig. 2b). Even though a repopulation process is observed in the muscle niche, its dynamic is different from that characterised in vitro (Fig. 2c). These observations suggested that SCA1-High and SCA1-Low-FAPs are dynamic states capable of repopulating each other with different dynamics in vitro and in vivo.

We next investigated whether SCA1-High-FAPs and SCA1-Low-FAPs could be distinguished in vitro according to their morphology. To this end, we stained their cytoplasms with CarboxyFluorescein Succinimidyl Ester (CFSE) and we selected three suitable descriptors to characterise cell shape^25^. Roundness and aspect ratio (AR) are parameters that describe round cells and elongated cells respectively (Supplementary Fig. 4f). The third descriptor is cell size, which was estimated by the CFSE positive area. As a control we included in this analysis a population enriched for MuSCs since they acquire characteristic shapes during myogenic differentiation. Indeed, in the MuSC sample we are able to identify cells that exhibit high roundness/low AR and cells with low roundness/high AR (Supplementary Fig. 4g–j). The former round cells are proliferating myoblasts, the latter elongated cells are differentiating myocytes^39^. Moreover, these myogenic cells are smaller than SCA1-High-FAPs or SCA1-Low-FAPs (Supplementary Fig. 4i). Focusing on SCA1-High-FAPs and SCA1-Low-FAPs, we found that these cells are indistinguishable from any of the three shape describing parameters thereby excluding cell morphology or size as grounds for the observed heterogeneity (Fig. 2d–g).

In conclusion, using as a criterion micro-heterogeneity in SCA-1 expression, we identified two FAP cell states that populate the skeletal muscle of the *mdx* mouse. The two cell states are occupied by morphologically identical cells and are dynamic as they readily lose their identity and acquire similar SCA-1 expression profiles.

### **S**CA1-High-FAPs have a higher adipogenic propensity than SCA1-Low-FAPs

The impact of macro-heterogeneity on the differentiation potential of adipose progenitors has been characterised in the adipose tissue^40^. Whether micro-heterogeneity also plays a similar role in adipogenic progenitors in the skeletal muscle, and in differentiation in general, remains unexplored. The FAP adipogenic potential is restrained in the skeletal muscle by negative signals. In pathological conditions, however, the several cues controlling this process are deregulated leading to the deposition of IMAT^19,20,41^. Muscles of the *mdx* mouse develop ectopic adipose tissue only in old animals^20^. However, when cultured ex vivo in the absence of the anti-adipogenic signals of the muscle niche, freshly isolated FAPs from young *mdx* mice readily differentiate into adipocytes^42^. We asked whether the micro-heterogeneity in the expression of SCA-1 has any impact on the ex vivo adipogenic potential.

**Fig. 3:**
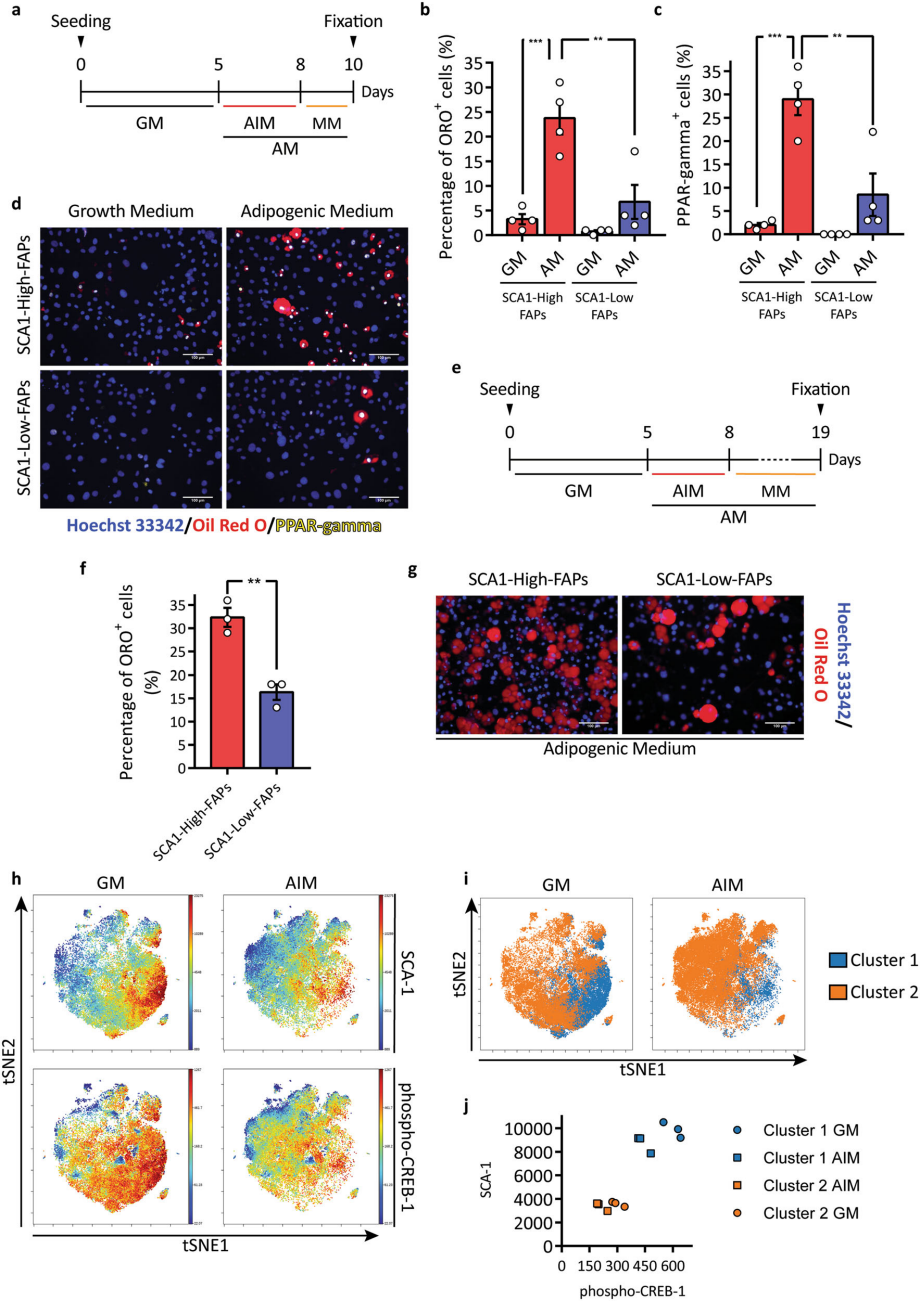
SCA1-High-FAPs differentiate more efficiently into adipocytes than SCA1-Low-FAPs. **a** Experimental design to induce adipogenic differentiation of *mdx* FAP cell states. GM = growth medium; AIM = adipogenic induction medium; MM = maintenance medium. **b** and **c** Bar plots showing the percentage of ORO positive cells and PPAR-gamma positive cells per field. AM = adipogenic medium (AM = AIM + MM). Statistical analysis was performed by a Two-way ANOVA (*n* = 4). **d** Representative micrographs of (**b**) and (**c**). Cells were immunolabelled for PPAR-gamma (yellow) and nuclei were counterstained with Hoechst 33342 (blue). Lipid droplets were stained with ORO (red). **e** Experimental design applied to obtain fully differentiated adipocytes. **f** Bar plot indicating the percentage of ORO positive cells (n = 3). Statistical significance was calculated through Student’s *t* test. **g** Representative micrographs of **(f)**. Lipid droplets in red (ORO staining) and nuclei in blue (Hoechst 33342). **h** Representative viSNE maps showing the expression of SCA-1 and phospho-CREB-1 assessed by mass cytometry in FAPs isolated from *mdx* mouse by MACS and cultured 72 h either in GM or AIM (*n* = 3). **i** Cluster 1 (in blue) and cluster 2 (in orange) obtained applying the FlowSOM algorithm and then mapped onto viSNE maps. **j** Plot representing the expression in arbitrary units of SCA-1 and phospho-CREB-1 identified in (**i**). Data are presented as mean ± SEM. **p ≤ 0.01, ***p ≤ 0.001. Scale bars 100 μm.

The two cell states of FAPs purified from *mdx*-mice muscles were cultured for 5 days in growth medium (GM). After this short expansion period, adipogenic differentiation was induced by incubating cells with the adipogenic induction medium (AIM), GM supplemented with 1 μg/ml insulin, 1 μM dexamethasone and 0.5 mM of 3-isobutyl-1-methylxanthine (IBMX), for three days followed by additional two days in the adipogenic maintenance medium (MM), GM supplemented with 1 μg/ml insulin (Fig. 3a). At the end of the differentiation process, we stained the lipid droplets using Oil Red O (ORO). We noticed little differentiation in both cell states when incubated in GM in the absence of adipogenic stimuli. Interestingly in cells incubated with the adipogenic medium (AM), the percentage of adipocytes in SCA1-High-FAPs is significantly higher than the one observed in SCA1-Low-FAPs (Fig. 3b, d). This different behaviour may be the result either of a different propensity to undergo adipocyte commitment or to a delay in adipogenic differentiation. We addressed this point by evaluating the expression of peroxisome proliferator-activated receptor gamma (PPAR-gamma) by immunofluorescence. This transcription factor is a master regulator of adipogenesis and is not expressed in FAPs soon after isolation^14,15^. PPAR-gamma expression is significantly higher in SCA1-High-FAPs (Fig. 3c, d) suggesting that adipocyte commitment may be impaired in SCA1-Low-FAPs. To further support this conclusion, we allowed a longer time for FAPs to differentiate into mature adipocytes by extending the incubation in MM to 11 days (Fig. 3e). This long-term insulin stimulation allows cells to display their full adipogenic potential. In this condition, both cell states differentiate more efficiently and adipocytes are more mature compared to what observed in the standard protocol (Fig. 3g). However, also in this condition, a larger percentage of SCA1-High-FAPs yielded mature adipocytes (Fig. 3f). At the end of the differentiation process, SCA1-High-FAPs reach a higher confluence when compared to SCA1-Low-FAPs (Supplementary Fig. 5a-b). To rule out the possibility that the observed differences in the adipogenic differentiation of FAP cell states could be a consequence of a different kinetics in reaching cell confluence, we plotted the total number of nuclei per field over the total number of adipocytes per field from the experiment in Fig. 3a–d and we calculated the trend lines^42^. Even though the trend line of SCA1-High-FAPs clearly reveals a correlation between cell confluence and adipogenic differentiation, the trend of SCA1-Low-FAPs shows no increment of adipocytes at increasing nuclei number (Supplementary Fig. 5c). This analysis is consistent with SCA1-Low-FAPS having lower adipogenic propensity, irrespective of cell confluence.

The correlation between SCA1 expression and adipogenic potential is also confirmed by a single-cell approach. FAPs were isolated from muscles of *mdx* mice by MACS and then were incubated in either GM or AIM. After 72 h single cells were analysed by mass cytometry using a panel of antibodies against 18 proteins (CD45, CD146, cleaved CASP-3, CD34, phospho-EGFR, CD140a, pRb, CD140b, Vimentin, CD90.2, phospho-STAT3, Integrin alpha-7, CXCR-4, SCA-1, CD31, c-Kit, Actin, phospho-CREB-1). viSNE maps highlight a correlation between phospho-CREB-1, a marker of early adipogenic differentiation, and SCA-1 expression in cells maintained in the growth medium (Fig. 3h). By applying the FlowSOM algorithm, we observed two clusters expressing high levels of SCA-1 and phospho-CREB-1 (the blue cluster) and low levels of these proteins (the orange cluster) (Fig. 3i, j). Consistently the levels of phospho-CREB-1 decrease in cells after three days of differentiation but remain higher in cells spontaneously differentiating in growth medium as CREB-1 phosphorylation is an early event in adipogenesis^43,44^. In addition, we found that SCA-1 expression decreases during the adipogenic differentiation of both bulk FAPs and SCA1-High-FAPs (Supplementary Fig. 5d).

Collectively, these results indicate that the different SCA-1 expression levels in the different FAP cell states correlate with a different adipogenic potential. SCA1-Low-FAPs are characterised by a reduced adipogenesis, whereas SCA1-High-FAPs engage in this differentiation path more readily.

### **F**AP cell states differ in the expression of fibrogenic genes

Abnormal deposition of extracellular matrix (ECM) is another pathogenic hallmark of muscular dystrophies. Differently from fat deposition this condition also develops in young dystrophic mice. Myofibroblasts differentiate from several cell types and, among these, FAPs are considered the main source of fibrotic scars^12^. Upon differentiation, myofibroblasts synthetise alpha-smooth muscle actin (alpha-SMA) to form positive stress-fibres and increase the production of ECM proteins^45^. Transforming growth factor beta-1 (TGF-beta-1) promotes fibrogenic differentiation of FAPs in vitro and takes part in fibrosis in vivo^12,46^.

**Fig. 4:**
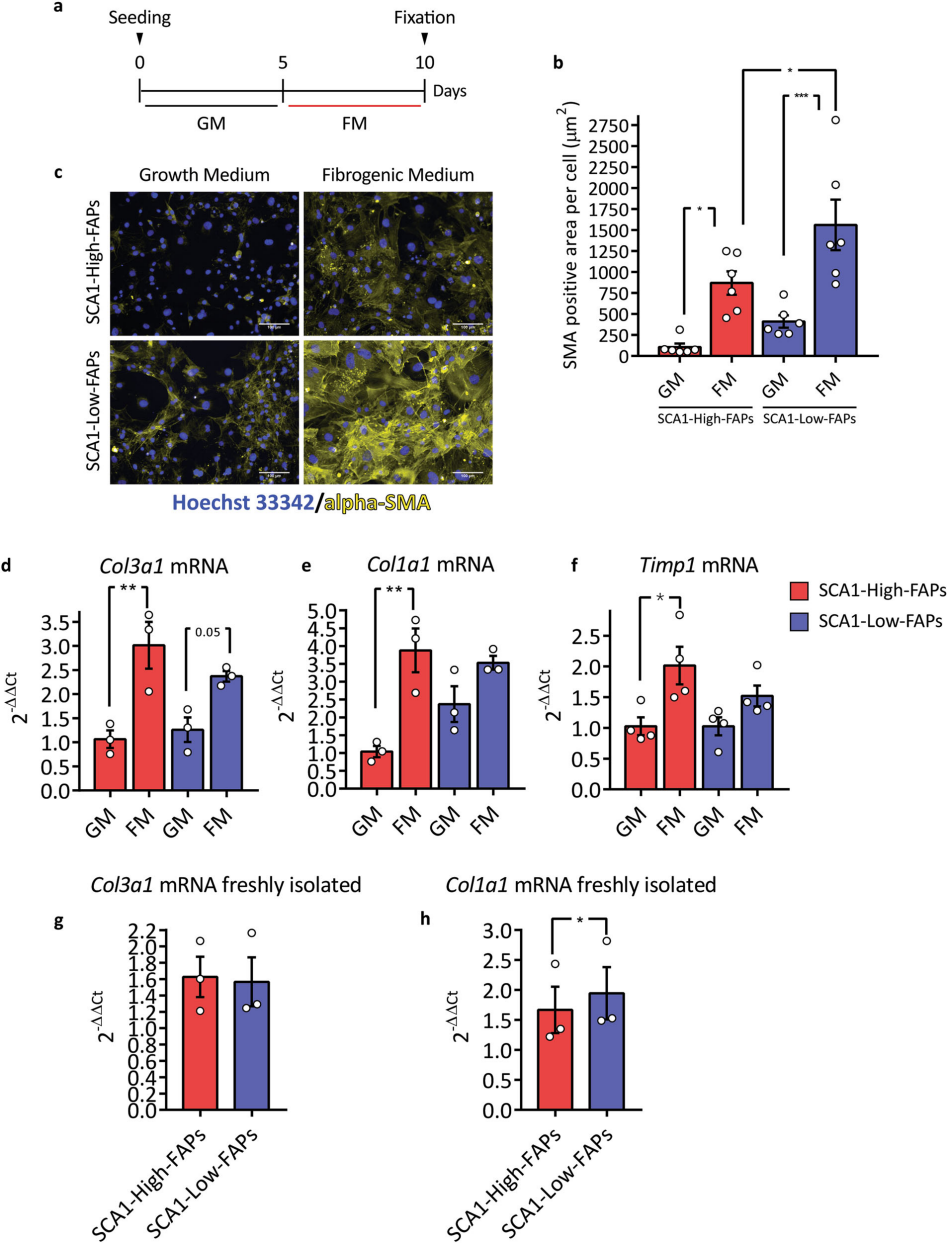
Fibrogenic differentiation of *mdx* FAP cell states. **a** Experimental design to induce fibrogenic differentiation of *mdx* FAP cell states. GM = growth medium; FM = fibrogenic medium. **b** Bar plot presenting alpha-SMA-positive area per cell in each field (n = 6). Statistical significance was evaluated using Two-way ANOVA. **c** Representative microphotographs of **(b**). Immunofluorescence against alpha-SMA (yellow) and nuclei counterstaining (blue, Hoechst 33342). Scale bar 100 μm. **d**–**f** Bar plots showing *Col3a1*, *Col1a1* (n = 3) and *Timp1* (n = 4) mRNA expression. Following 5 days of differentiation samples were analysed by Real-Time PCR. Statistical significance was estimated through a Two-way ANOVA. **g**, **h** Bar plots showing *Col3a1* and *Col1a1* mRNA expression immediately after sorting (n = 3). Samples were analysed by Real-Time PCR using 2^-ΔΔCt^ comparative method. *Tubulin* mRNA was set as reference gene. Statistical significance was evaluated by Student’s *t* test. Data are presented as mean ± SEM. *p ≤ 0.05, **p ≤ 0.01, ***p ≤ 0.001.

We asked whether FAP heterogeneity, in addition to modulating adipogenesis, could also impact on fibrogenesis. After 5 days in growth medium (GM), FAPs were exposed for 5 additional days to a fibrogenic medium (FM) containing TGF-beta-1 (Fig. 4a). Next, the expression of alpha-SMA was monitored by immunofluorescence. Both cell states respond to FM by increasing the alpha-SMA-positive area. The alpha-SMA-positive area surrounding SCA1-Low-FAP cells was twice as large as that of SCA1-High-FAP cells (Fig. 4b, c, nuclei count in Supplementary Fig. 5e). The SMA-positive area in samples exposed only to GM also tends to be higher in SCA1-Low-FAPs.

We also monitored the mRNA levels of *Col3a1* and *Col1a1* genes upon fibrogenic induction. These collagen chains are components of the skeletal muscle ECM and are produced by FAPs upon TGF-beta-1 treatment in vitro^12^. Both cell states respond to FM by increasing the expression of *Col3a1* mRNA (Fig. 4d). However, only SCA1-High-FAPs significantly increased the expression of *Col1a1* mRNA upon induction of differentiation (Fig. 4e). By looking at the mRNA levels in the absence of induction of fibrogenesis (GM), SCA1-Low-FAPs tend to express higher level of *Col1a1*. This difference was significant in freshly isolated cells (Fig. 4h). We found no difference in *Col3a1* mRNA levels when the two cell states were compared in freshly isolated cells or after growth in GM (Fig. 4g). Moreover, we monitored the expression of the *Timp1* mRNA, another transcript expressed by FAPs upon in vitro fibrogenic stimulation^12^. SCA1-High-FAPs but not SCA1-Low-FAPs respond to the FM enhancing their expression of the *Timp1* mRNA (Fig. 4f).

We conclude that both FAPs cell states are able to differentiate into myofibroblasts when exposed to a fibrogenic microenvironment, but they exhibit a differential expression of at least two genes coding an ECM component.

### **S**CA1-High-FAPs are characterised by a transcriptional profile responsive to adipogenic stimuli

**Fig. 5:**
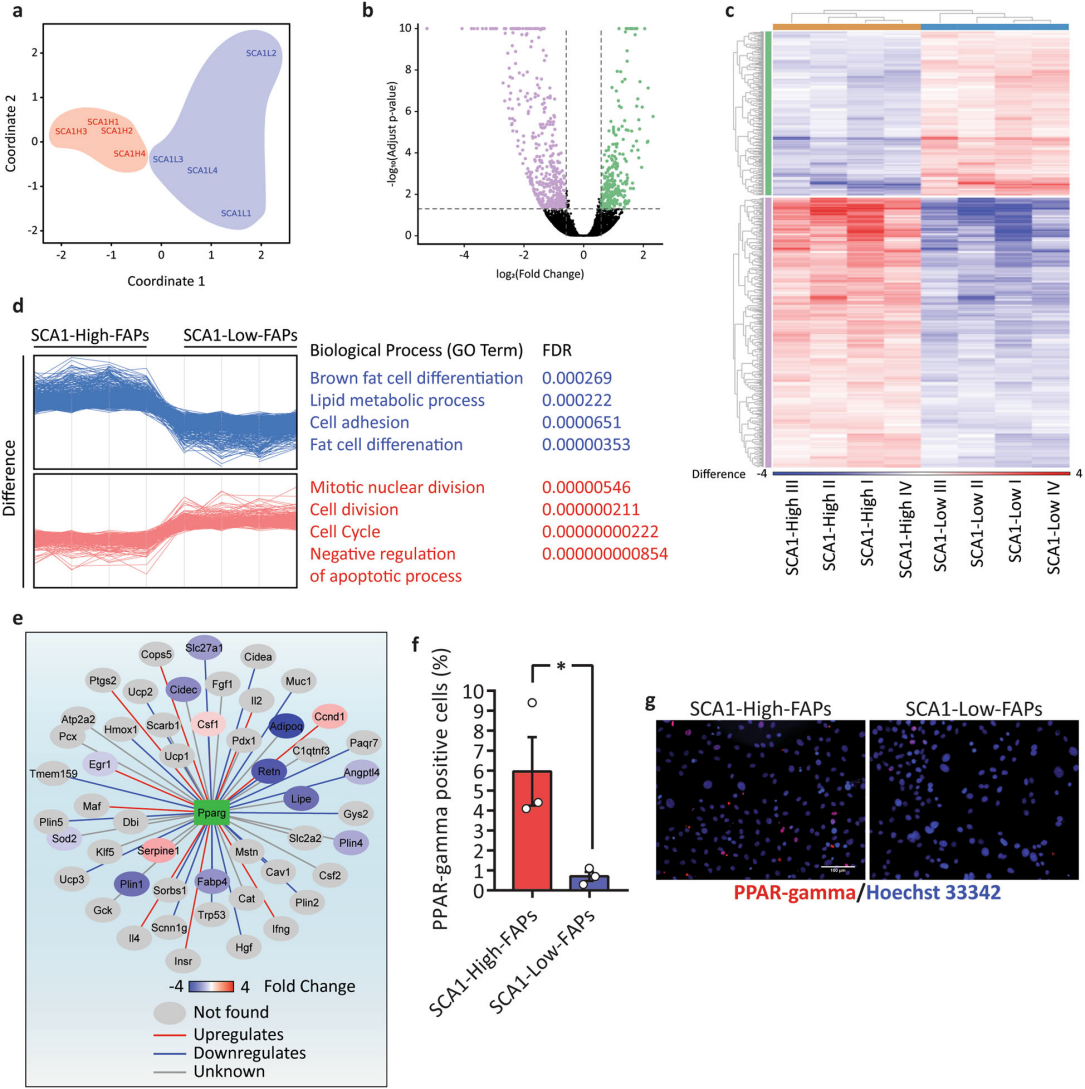
SCA1-High-FAPs have a transcriptional profile committed to the adipogenic differentiation. **a** Multidimensional scaling (MDS) plot of FAP cell states transcriptome. **b** Volcano plot of log10(adjust *p*-value) versus log2(fold change) showing upregulated (in green) and downregulated (in purple) genes in SCA1-Low-FAPs. **c** Heatmap of the Difference of the different biological replicates (*n* = 4). **d** Profile plots of the Difference of the downregulated (upper panel, purple cluster in Fig. 5c) and upregulated genes (lower panel, orange cluster in Fig. 5c) in SCA1-Low-FAPs. The right panel shows the top 4 Biological processes GO terms significantly enriched (FDR < 0.05) for upregulated and downregulated genes in SCA1-Low-FAPs. **e** A network in which each node represents a gene transcriptionally regulated by PPAR-gamma (from TRRUST). Red edges: genes activated by PPAR-gamma; blue edges: genes repressed by PPAR-gamma; grey edges: unknown mechanism. The nodes are filled according to the Fold Change. **f** Bar plot showing the percentage of PPAR-gamma positive cells after 5 days in GM (*n* = 3). **g** Representative images of bar plot in the previous panel. PPAR-gamma in red and nuclei in blue. Scale bar 100 μm. Statistical analysis was performed with Student’s *t* test. Data are presented as mean ± SEM. *p ≤ 0.05.

We next set out to compare the transcriptional landscapes of the two FAP cell states as it may shed light on the molecular determinants of their different responsiveness to adipogenic and fibrogenic stimuli. Therefore, we studied their transcriptional profiles by RNA-sequencing after 5 days of incubation in GM. The multidimensional scaling (MDS) plot of the transcriptional data shows that the two cell states at this time, before differentiation induction, have distinct transcriptomes (Fig. 5a), a difference which is most noticeable along coordinate 1. The differentially expressed genes are 704 (adjusted *p*-value < 0.05) 266 of which are up regulated (log2 fold change > +0.5) and 438 are downregulated (log2 fold change < −0.5) in SCA1-Low-FAPs (Fig. 5b). Samples are grouped into 2 different clusters that discriminate SCA1-High-FAPs (the orange cluster) and SCA1-Low-FAPs (the blue cluster) (Fig. 5c). Moreover, differentially expressed genes are grouped into 2 clusters of upregulated and downregulated genes (Fig. 5c, d). A gene set enrichment analysis of the “Biological Process” GO terms revealed that in SCA1-High-FAPs the genes involved in adipocyte differentiation (GO terms “brown fat differentiation” and “fat cell differentiation”), among others, are over-represented in the list of upregulated genes (Supplementary Table 4) (Fig. 5d). This is consistent with the observation that SCA1-High-FAPs are more prone to differentiate into adipocytes. In agreement with this hypothesis, some of the genes that are transcriptionally regulated by PPAR-gamma are upregulated in SCA1-High-FAPs (Fig. 5e) and PPAR-gamma is more expressed in SCA1-High-FAPs (Fig. 5f, g).

The second most upregulated mRNA in SCA1-High-FAPs is *Adipoq* which encodes for adiponectin (log2 fold change = −4.07). We confirmed this differential expression by Real-Time PCR (Supplementary Fig. 6a). Adiponectin is a well-known sensitiser of the insulin pathway^47,48^. For this reason, we hypothesise that the spontaneous adipogenic propensity of SCA1-High-FAPs is likely due to an adiponectin-mediated autocrine circuit, which is not active in SCA1-Low-FAPs (Supplementary Fig. 6b). According to this hypothesis, adiponectin sensitises SCA1-High-FAPs to the adipogenic mix which contains insulin. Indeed, a 24 h pre-treatment of SCA1-Low-FAPs with AdipoRon, a small molecular which binds and activates the adiponectin receptors^49^, induces a three-fold increase of their sensibility to the adipogenic mix (Supplementary Fig. 6c, d).

Regarding fibrogenic differentiation, we found that several collagen genes are differentially regulated. Among these, the *Col3a1* mRNA is upregulated in SCA1-High FAPs (log log2 fold change = −1.03), while *Col12a1* mRNAs are upregulated in SCA1-Low-FAPs (log2 fold change = 0.82). Consistent with the observation that SCA-1 activates metalloproteinases (MMPs) expression^50^, three MMPs genes are upregulated in SCA1-High-FAPs: *Mmp11* (log2 fold change = −0.84), *Mmp23* (log2 fold change = −0.91) and *Mmp19* (log2 fold change = −0.81) (Supplementary Fig. 6e, g). Finally, SCA1-High-FAPs express higher levels of *Alpl* mRNA than SCA1-Low-FAPs (Supplementary Fig. 6h).

In conclusion, the two FAP cell states differentially express several genes that participate in ECM assembly and remodelling. At the same time, SCA1-High-FAPs spontaneously acquire a transcriptional profile that sensitise them to induction of adipogenic differentiation, possibly under an autocrine signalling circuit mediated by adiponectin.

### **S**CA1-High-FAPs proliferate more extensively than SCA1-Low-FAPs

A coordinated modulation of FAP proliferation and apoptosis is a key requisite to achieve efficient muscle regeneration^17^. Indeed, the deregulation of these processes observed in DMD leads to FAP accumulation and the ensuing deposition of ectopic tissues^21^.

**Fig. 6:**
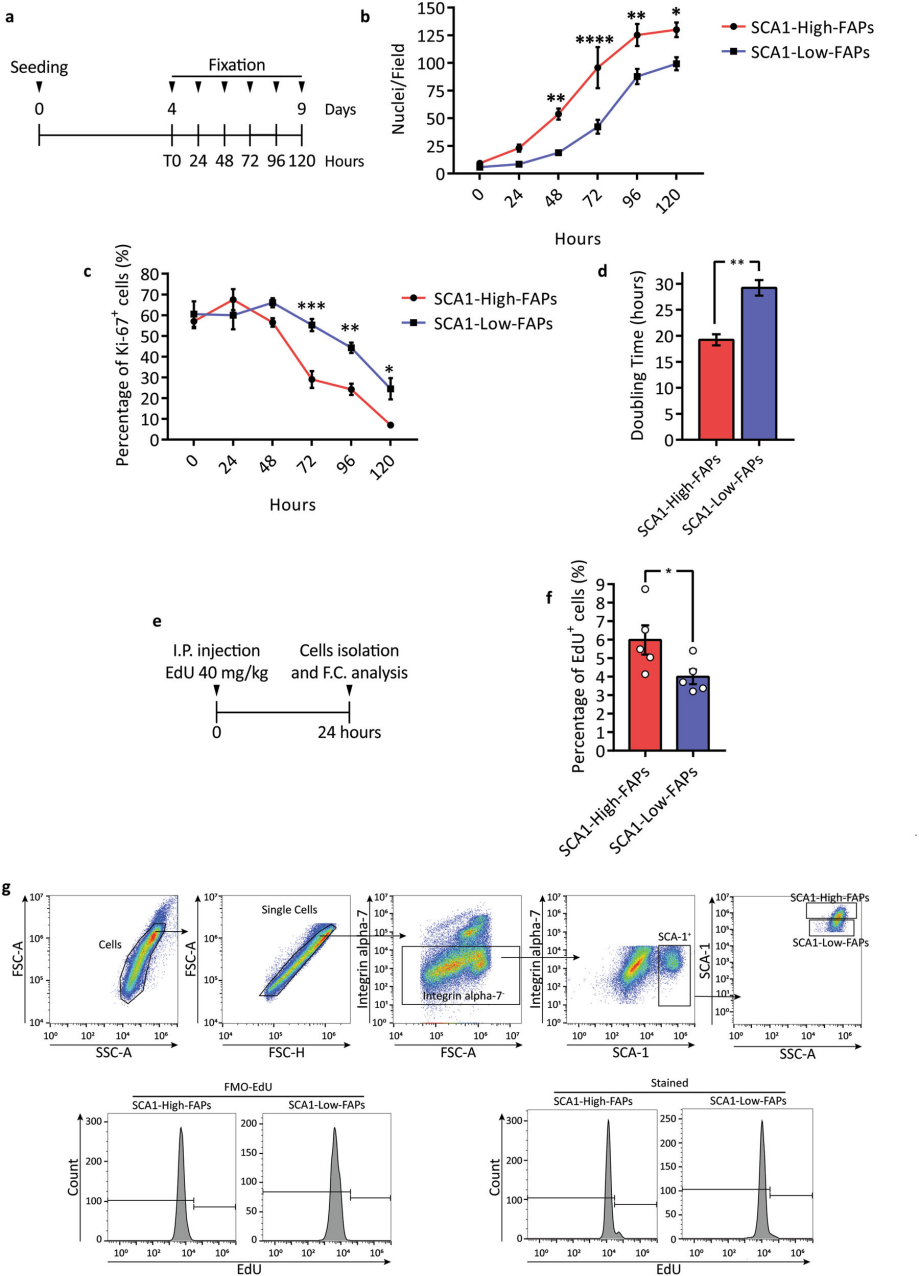
SCA1-High-FAPs have a higher proliferation rate than SCA1-Low-FAPs. **a** Schematic representation of the experimental plan to study *mdx* FAP cell states proliferation rate in vitro. Collected samples were immunolabelled for Ki-67 and nuclei were counterstained with Hoechst 33342 (*n* = 4). **b** Growth curve showing the number of nuclei per field. **c** Plot representing the percentage of Ki-67 positive nuclei per field. **d** Doubling time of FAP cell states calculated using the first three days of exponential growth. Student’s *t* test was applied to determine statistical significance. **e** Experimental plan applied to investigate cell states proliferation in vivo. EdU at the concentration of 40 mg/kg was injected into the peritoneum of *mdx* mice. After 24 h mice were sacrificed and EdU incorporation was studied by flow cytometry. **f** Bar plot showing the percentage of FAP cell states in proliferating FAPs (*n* = 5). **g** Gating strategy to evaluate EdU incorporation. For the unstained sample and FMO controls see Supplementary Fig. 7b, c. Data are presented as mean ± SEM. Statistical analysis was performed using Two-way ANOVA for the in vitro experiment and Student’s *t* test for the in vivo experiment. * *p* ≤ 0.05, ** *p* ≤ 0.01, **** *p* ≤ 0.0001.

We investigated whether the observed FAP heterogeneity would also affect cell proliferation. Cells were sorted from *mdx* mice and the two cell states were plated at the same low density. Starting at the first time point (T0), we collected samples every 24 h (Fig. 6a) and we counted the number of nuclei and the percentage of Ki-67 expressing cells to identify proliferating cells. Already at 48 h, the number of SCA1-High-FAPs nuclei in a field is significantly higher than in the SCA1-Low-FAPs (Fig. 6b). However, the percentage of cells expressing Ki-67 is comparable in freshly purified cells and decreases more rapidly at later times in SCA1-High-FAPs (Fig. 6c) as this cell state reaches confluence earlier and stops growing. In the exponential growth phase, the doubling time of SCA1-High-FAPs is about 10 h shorter (Fig. 6d and Supplementary Fig. 7a). Taken together these results suggest that ex vivo SCA1-High-FAPs have a shorter duplication time reaching at late time points a higher confluence and a lower number of Ki-67 positive cells.

In order to lend support to an in vivo relevance of these observations, we injected 5-ethynyl-2′-deoxyuridine (EdU) into the peritoneum of *mdx* mice. After 24 h we isolated mononuclear cells (Fig. 6e) and we selected CD45^−^ CD31^−^ cells by magnetic activated cell sorting (MACS). Next, we identified FAP cell states in the Integrin alpha-7^−^ SCA-1^+^ compartment and monitored EdU incorporation using flow cytometry (Fig. 6g; for the controls refer to Supplementary Fig. 7b, c). A larger fraction of SCA1-High-FAPs have incorporated EdU in comparison with SCA1-Low-FAPs (6% versus 4%, *p*-value < 0.05) (Fig. 6f). In conclusion, we observed a difference in the proliferation rate of FAP cell states in vitro which correlates with a higher fraction of SCA1-High-FAPs incorporating EdU in vivo.

### Single-cell transcriptomic analysis of FAP cell states from wild type mice

**Fig. 7:**
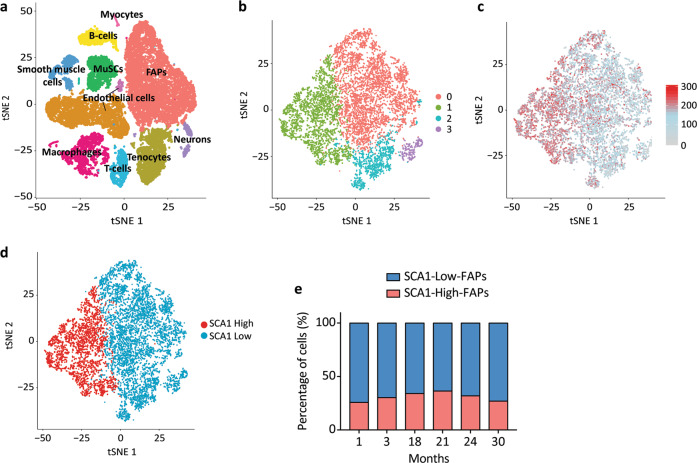
FAP cell states from wild type mice differ in their transcriptional profile. **a** viSNE map representing the different clusters identified as distinct cell populations according to the expression of specific biomarkers. Identified populations are: myocytes, B-cells, FAPs, smooth muscle cells, MuSCs, endothelial cells, macrophages, T-cells, tenocytes and neurons. **b** The FAPs population subset has been re-clustered, leading to 4 distinct clusters according to their gene expression profile. The four clusters (0, 1, 2,3) were mapped onto the viSNE map of FAPs. **c** The expression of SCA-1 antigen has been mapped to each cell of the dataset. **d** viSNE map of FAPs in which clusters 0, 2, 3 are collapsed together to obtain two clusters expressing high level (SCA-1 High) and low level (SCA-1 Low) of SCA-1. **e** Stacked bar plot showing the percentage of the two population on the basis of SCA-1 expression levels across different ages.

Till now we have used the expression of a single antigen (SCA-1) to characterise two FAP cell states displaying distinct differentiation and proliferation characteristics. In these experiments, cells were purified from muscles of *mdx* mice as in this model the two cell states are more evenly represented. However, heterogeneity in the expression of SCA-1 is observed both in wild type and *mdx* muscles (Fig. 1). To obtain a more general picture of the observed heterogeneity by an independent experimental approach we made use of the Tabula Muris Senis dataset^51^. The Tabula Muris Senis is a comprehensive compendium of single-cell RNA-sequencing datasets in 23 organs from wild type mice at different ages. We focused on the skeletal muscle and performed an unbiased automated clustering using markers from the Myo-REG resource (Supplementary Fig. 8a) to identify the main skeletal muscle populations^52^. We obtained 11 clusters (Fig. 7a) with an expression profile characteristic of each population (Supplementary Fig. 8b–l). Next, we carried out an additional clustering analysis on the FAP population and observed four clusters (Fig. 7b) characterised by different levels of SCA-1 expression (Fig. 7c). Cluster 0 (red), cluster 2 (blue) and cluster 3 (purple) express similar low level of SCA-1 and where collapsed into a single group, thus obtaining two populations expressing high and low SCA-1 levels (Fig. 7d). SCA1-Low-FAPs are more numerous than SCA1-High-FAPs (Fig. 7e), similarly to what was observed in young mice using the flow cytometry assay (Fig. 1c). Moreover, their ratio only slightly increases in ageing.

We performed a Gene Ontology (GO) term enrichment analysis on differentially expressed genes which did not provide any robust clue regarding FAP cell states properties. However, the list of differentially expressed genes revealed that SCA1-High-FAPs express higher levels of *Dpp4* mRNA which is a marker of mesenchymal progenitors residing in the reticular interstitium of inguinal white adipose tissue (iWAT)^40^. In this tissue, Dpp4 characterise progenitors able to differentiate into adipocytes suggesting an analogy between such progenitors and SCA1-High-FAPs. Next, we asked if the differential expression of the *Col1a1* gene observed in *mdx* FAP cell states (Fig. 4) is conserved in the wild type counterpart. *Col1a1* mRNA is slightly downregulated in SCA1-High-FAPs (average log_2_ fold change = -0.341, adjusted *p*-value < 0.05), in accord with our findings in FAP cell states freshly isolated from *mdx* mice.

We conclude that single-cell transcriptional profiling of wild type FAPs allow us to distinguish two cell states on the basis of SCA-1 transcriptional levels. The analysis of genes that are differentially expressed in the two cell states provides hints about possible mechanisms underlying the observed phenotypic differences.

### **S**ignals secreted from cells of the immune compartment control adipogenesis of FAP cell states in an age-dependent fashion

In vivo the FAP proliferation and differentiation traits are controlled by the muscle microenvironment^14,15^. The prolonged and sustained chronic inflammation of *mdx* muscles is characterised by a subset of unconventional macrophages. It was shown that in an in vivo model recapitulating muscle aging in *mdx* mice, these macrophages fail to induce FAP apoptosis and promote their excessive growth^21^. Others report that, in wild type mice, ageing determines a decrease in the number of FAPs associated with a reduced adipogenic potential^53^.

**Fig. 8:**
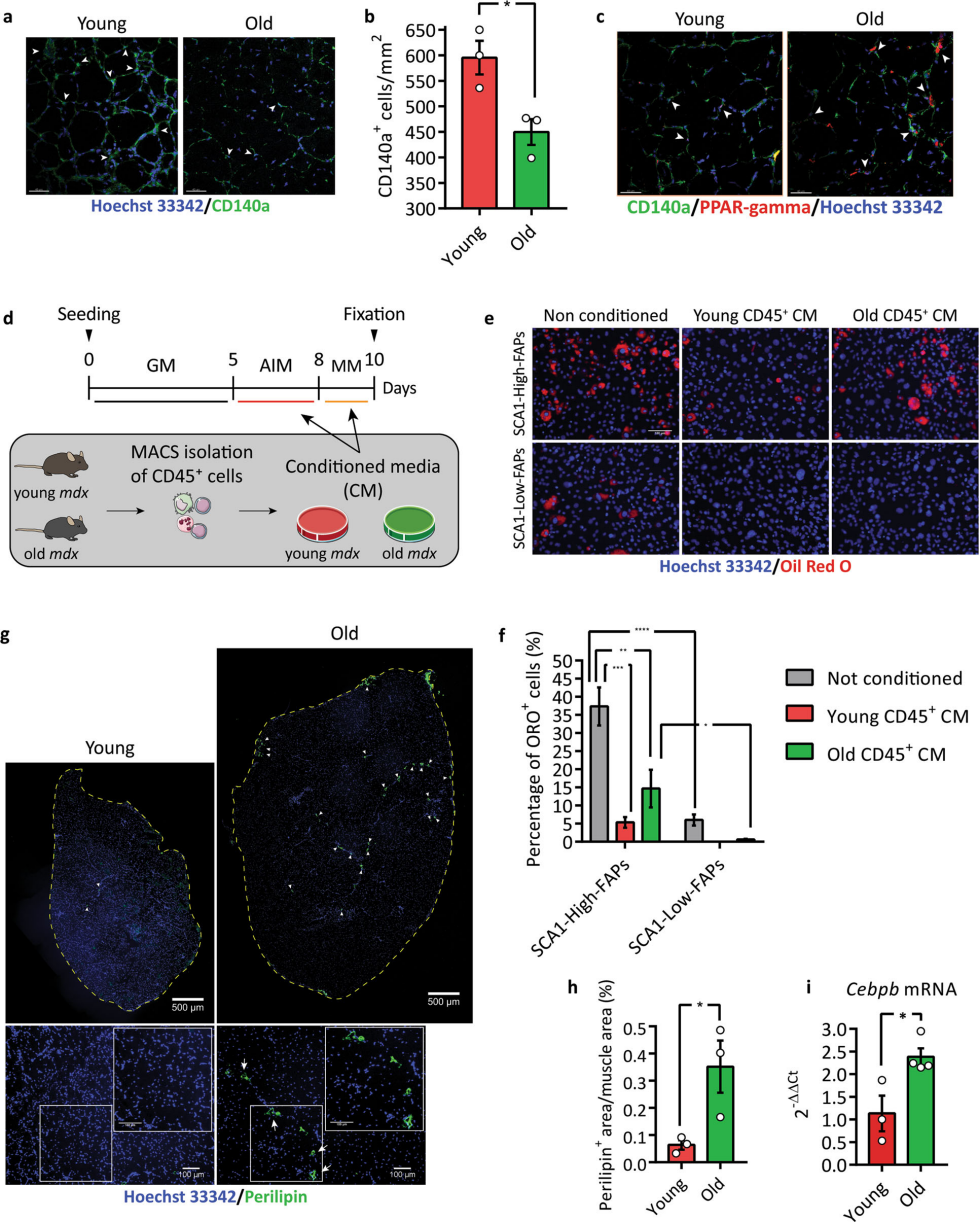
Immune system from old *mdx* mice releases adipogenic potential of SCA1-High-FAPs. **a** Representative images of the experiment whose results are reported in the bar plot in (**b)**. FAPs were identified by an antibody against CD140a (green) and nuclei were counterstained with Hoechst 33342 (blue). Scale bar 40 μm. **b** Bar plot representing the number of FAPs (CD140a^+^ cells) per mm^2^ in a section of TA from young and old *mdx* mice. Statistical analysis was carried out by Student’s *t* test (*n* = 3). **c** Representative immunofluorescence of TA section of young and old mdx mice showing PPAR-gamma positive FAPs. FAPs were immunolabelled with an antibody against CD140a (green) and nuclei were counterstained with Hoechst 33342 (blue). In red cells expressing PPAR-gamma. Scale bar 40 μm (*n* = 3). **d** Schematic representation of the experiment in (**e**) and (**f**). Leucocytes (CD45^+^ cells) were isolated through MACS from young and old *mdx* mice. After 24 h of ex vivo culture their conditioned media (CM) were harvested and used to induce adipogenesis of young FAP cell states from *mdx* mice in combination with AIM and MM. Cells and dish are taken from Servier Medical Art (SMART), under Creative Commons Attribution 3.0 Unported License. **e** Representative microphotographs of **(d)**. Lipid droplets were stained with ORO (red) and nuclei with Hoechst 33342 (blue). **f** Bar plot showing the percentage of ORO positive cells (n = 3). Statistical significance was evaluated using Two-way ANOVA. Scale bar 100 μm. **g** Immunofluorescence against perilipin on TAs from young (left) and old (right) *mdx* mice. Representative images of reconstruction of the whole TA section. Perilipin is showed in green and nuclei in blue. Dashed lines highlight section borders. Scale bar 500 μm. Lower insets show images used to reconstruct the whole muscle. Scale bar 100 μm. Arrows indicate perilipin positive cells. **h** Bar plot presenting the percentage of Perilipin positive area in the whole TA section. Statistical significance was evaluated using Student’s *t* test. *n* = 3. **i** Bar plot showing the expression of *Cebpb* mRNA in the TA of young (*n* = 3) and old (*n* = 4) *mdx* mice assessed by Real-Time PCR using 2^-ΔΔCt^ comparative method. *Tubulin* mRNA was set as reference gene. Statistical significance was evaluated with Student’s *t* test. Data are presented as mean ± SEM. *p ≤ 0.05, **p ≤ 0.01, ***p ≤ 0.001, ****p ≤ 0.0001.

To find out how ageing affects FAPs and FAP cell states in the *mdx* mouse we determined the number of FAPs in the TA of young and aged dystrophic mice by immunofluorescence (Fig. 8a, b). We observed a reduction in the number of FAPs within old dystrophic muscles and this condition is followed by an increase of FAPs expressing PPAR-gamma in the tibialis anterior (TA) of aged *mdx* mice (Fig. 8c). These results suggest that FAPs in the microenvironment of aged *mdx* mice are more committed to the adipogenic differentiation. To verify this hypothesis we set out to investigate the effect of the immune cell compartment of young and old *mdx* mice on the adipogenesis of FAP cell states. For this purpose, we isolated CD45^+^ cells (henceforth called leucocytes) from young (6–8 weeks) and old (60 weeks) *mdx* mice and after 24 h of in vitro culture we harvested their supernatants. Next, we added each of these conditioned media (CM) to AIM and MM to test their effect on young FAP cell states differentiation (Fig. 8d). Adipocytes were identified by ORO staining. The CM of leucocytes from young *mdx* mice significantly inhibits adipogenesis of SCA1-High-FAPs (~7-fold decrease) (Fig. 8e, f). We observed a less important inhibition using the medium conditioned by leucocytes from old *mdx* mice (~3-fold decrease). In general, the differentiation potential of FAPs in both cell states is inhibited by the supernatants conditioned by leucocytes from young *mdx* mice and less so by supernatants of leucocytes from old *mdx* mice. While in these conditions the differentiation of SCA1-Low-FAPs is cut down to negligible levels, the differentiation of SCA1-High-FAPs still remains significant especially when exposed to conditioned media of leucocytes from old *mdx* mice. These observations suggest that the microenvironment of young *mdx* mice is more efficient in inhibiting FAP adipogenesis and probably sufficient to keep in check the differentiation of both FAP cell states. With aging, the negative control exerted by the CD45^+^ cell compartment becomes less effective, selectively releasing the adipogenic potential of SCA1-High-FAPs. These findings offer a possible mechanism to explain the appearance of IMAT in the TA of aged *mdx* mice as demonstrated by the increase of immunostaining against Perilipin (Fig. 8g, h) and the increase of *Cebpb* mRNA in the whole TA (Fig. 8i).

## Discussion

Non-genetic heterogeneity in clonal cell populations is suggested to underlie fundamental developmental and pathological processes^7,37,54,55^. However, as a consequence of the dearth of appropriate technologies, this concept has not been widely addressed as many experimental approaches still rely on bulk analysis of cell populations. The emergence, over the past decade, of powerful single-cell approaches has permitted to uncover a common hidden heterogeneity in biological systems^56^. To address the relation between heterogeneity and disease, we applied a multiplex flow cytometry approach to study the expression of nine surface antigens in a suspension of primary cells from a solid tissue. The single-cell expression profiles were used in a clustering approach to identify fibro/adipogenic progenitors (FAPs) in wild type and dystrophic mice. We confirmed the inherent heterogeneity of this cell population (Supplementary Fig. 2) and by focusing on the expression of the SCA-1 antigen we identified two cell clusters that are present with different abundance in the muscles of wild type and *mdx* mouse models. Interestingly an unsupervised clustering of the single-cell gene expression dataset of the Tabula Muris Consortium also leads to similar conclusions identifying clusters of FAPs characterised by different expression levels of SCA-1. To characterise FAP micro-heterogeneity we have used flow cytometry to monitor the expression of FAP surface markers. However, as our antibody panel was limited, the combination of surface markers used in our analysis is not sufficient to clearly distinguish all the interstitial populations involved in muscle regeneration, such as for instance mesoangioblasts (MABs)^57^, Pw1^+^/Pax7^+^ interstitial cells (PICs)^58^ and Twist2^+^ progenitors^59^. The 11 unassigned clusters (Supplementary Fig. 1d) that we did not pursued in our analysis may contain these cell types. In addition, the SCA-1 marker is not unique for FAPs, as it is also expressed by mesoangioblasts (MABs)^57,60,61^. As single-cell RNA-seq analysis also indicates a degree of overlap between MABs and FAPs we wanted to exclude that our cell preparation contained a mixture of FAPs and MABs^61^. To further characterise our preparation, we monitored the expression of alkaline phosphatase, a marker of the perivascular niche which includes MABs and pericytes. While we observed that both cell types express alkaline phosphatase activity, we noticed that SCA1-High-FAPs express higher level of *Alpl* mRNA. However, the cells in our FAP preparation do not have myogenic potential, a feature that characterise type 2 pericytes and MABs^62^. In addition they express high level of PDGFR-alpha, a surface marker that differentiate FAPs from MABs and pericytes^14,16^.

Different kinds of cell to cell variability within a monoclonal cell population have been described using single-cell technologies. In particular, the term micro-heterogeneity refers to a condition in which the distribution of the expression of a given trait, an mRNA or a protein, in a cell population is represented by a bell-shaped curve whose standard deviation is larger than expected by random noise or experimental error^6,63^. We identified such a condition in the expression distribution curve of SCA-1 in FAPs. SCA-1 micro-heterogeneity was already shown to modulate lineage choice in a Erythroid myeloid lymphoid (EML) cell line^7,64^. This consideration encouraged us to further investigate the role of SCA-1 heterogeneity in fate decision of dystrophic FAPs. To this purpose, based on the results of our multiplex flow cytometry approach and prior knowledge^7,8,37^, we resolved the continuity of the distribution of SCA-1 expression, a process defined decomposition^63^, into two discrete groups of cells by a fluorescence activated sorting strategy based on SCA-1 expression levels.

Although efforts have been made, clear definitions and terminologies regarding sub-populations and cell states are still lacking. Here we use two in vitro criteria to distinguish these conditions: the nature of their heterogeneity and the ability to maintain their identity once they are separated and cultured in vitro. By applying these criteria, the two groups of dystrophic FAPs were found to be morphologically identical cell states able to repopulate each other in vitro. Although we have shown that also in vivo FAP cell states are not static and repopulate each other to a certain degree, the dynamics and the final equilibrium of such a process is affected by the muscle microenvironment. The extent of this repopulation and its effect on in vivo FAP lineage choice as well as the signals from the niche that determine its dynamics remain to be established.

The involvement of micro-heterogeneity in the decision of cell fate has been addressed in different biological contexts. In the pre-implantation embryo, blastomeres express variable level of CDX2 (Ref. ^55^). In HeLa cells, the probability and timing of cell death in response to TRAIL are determined by the variance in protein levels of its signalling cascade^54^. The role of stem cell heterogeneity during muscle regeneration was recognised only lately^8^ and its contribution to FAP phenotype in Duchenne muscular dystrophy (DMD) has been highlighted even more recently^18^. However, its role in the development of ectopic tissues in DMD is unexplored. Here we have reported our investigation on the impact of the observed FAP heterogeneity on differentiation and proliferation in FAPs isolated from the muscles of the *mdx* mouse, an animal model of DMD^65^. FAPs are exposed in vivo to a complex and dynamic microenvironment which adds further complexity^66^, hence initially we performed our investigation in vitro to take into account only their intrinsic SCA-1 heterogeneity and their response to a controlled microenvironment. We demonstrated that SCA-1 micro-heterogeneity affects the expression of PPAR-gamma in response to an adipogenic medium resulting in a higher propensity of SCA1-High-FAPs to fully differentiate into adipocytes. Moreover, despite FAP cell states showing a similar propensity to differentiate into myofibroblasts, SCA1-Low-FAPs do not increase their expression of *Col1a1* and *Timp1* mRNAs after fibrogenic stimulation. Interestingly, these cells express higher amount of *Col1a1* mRNA in both *mdx* and wild type mice. We surmise that the expression of *Col1a1* gene in SCA1-Low-FAPs do not respond to the fibrogenic medium probably because of an already high basal transcription. Analysis of the transcriptional profile of SCA1-High-FAPs revealed that they preferentially express genes that are likely to make them more sensitive to adipogenic induction by insulin and uncover a possible pro-adipogenic autocrine circuit mediated by adiponectin. We also checked if SCA-1 micro-heterogeneity has an influence on FAP proliferation, a central process in muscle regeneration. The increase of approximately 10 h in the doubling time of SCA1-Low-FAPs decrease their proliferation rate and lengthen the period in which cells are in proliferation. Overall, our results demonstrate that micro-heterogeneity in SCA-1 expression is involved in the fate decision of dystrophic FAPs in a controlled in vitro environment and raise the possibility that this type of cell to cell variability may play a role in the differentiation and proliferation of mesenchymal-like cells in other pathological contexts.

What is known of the function of SCA-1 is not sufficient to rationalise the observed difference in the differentiation potential of the SCA-1-High and SCA-1-low cell states. Alternatively, and more likely, SCA-1 micro-heterogeneity only correlates with the observed phenotypes without being their direct cause. The molecular cause of micro-heterogeneity was attributed to a transcriptome-wide noise originating via a plethora of mechanisms^6,7,64^. We have used SCA-1 expression mainly as a tool to identify FAPs with divergent phenotypes. A future challenge is the clarification of the origin of this heterogeneity. An additional challenge consists in the elucidation of the physiological relevance of these observations in vivo where further complexity is added by the interaction of micro-heterogeneity with a dynamic microenvironment. To provide some evidence in this respect, we analysed the proliferation of FAPs by looking at the incorporation of EdU after injection into the peritoneum of *mdx* mice. The results confirmed that in vivo, as in isolated cells, FAPs expressing different level of SCA-1 proliferates to a different extent.

Our results show an increase of Perilipin and *Cebpb* mRNA in old *mdx* mice muscles associated with a decreased number of FAPs. These FAPs are also more committed to adipogenic differentiation as demonstrated by the increase of PPAR-gamma positive FAPs. We considered the possibility that the microenvironment under the stress caused by the muscle chronic damage could affect FAP micro-heterogeneity and differentially promote or inhibit the adipogenic potentials of FAP expressing high and low levels of SCA-1. To this end, we repeated the differentiation experiment using adipogenic media conditioned by leucocytes (CD45^+^ cells) from young and old *mdx* mice. In this condition, the two FAP cell states respond differently to the stimuli in the leucocyte supernatants. Adipogenesis of both cell states is inhibited by leucocyte secreted factor(s). The supernatant of leucocytes from young *mdx* mice is more efficient in restraining adipogenesis of both FAP cell states consistent with the observation that no fat infiltrations are observed in young *mdx* mice. With aging, however, leucocytes become less efficient in inhibiting adipogenesis of SCA1-High-FAPs. These conditions are still far from closely matching the in vivo microenvironment of young and old dystrophic muscles, as for instance the regulatory mechanisms provided by the muscle fibres are not included^19^. However, these observations, suggesting a different age-dependent response of FAP cell states to the anti-adipogenic signals of the immune compartment may explain the increase of IMAT in muscles of old dystrophic mice.

In conclusion, we surmise that micro-heterogeneity in SCA-1 expression plays a role during the decision-making of *mdx* FAPs in vitro and it is affected by the environment of dystrophic muscles.

## Supplementary information

Supplementary Information

Supplementary table 1

Supplementary table 2

Supplementary table 3

Supplementary table 4

Supplementary figure 1

Supplementary figure 2

Supplementary figure 3

Supplementary figure 4

Supplementary figure 5

Supplementary figure 6

Supplementary figure 7

Supplementary figure 8

## Data Availability

The RNA-seq data have been deposited in GEO with the identifier GSE163016.

## Code availability

The code for single-cell RNA-seq analysis is available in GitHub at the following link: https://github.com/Alessandro-Palma/Sca1/blob/main/analyse.R.

## References

[1] Bianconi E (2013). An estimation of the number of cells in the human body. Ann. Hum. Biol..

[2] Spitzer MH, Nolan GP (2016). Mass cytometry: single cells, many features. Cell.

[3] Macosko EZ (2015). Highly parallel genome-wide expression profiling of individual cells using nanoliter droplets. Cell.

[4] Buettner F (2015). Computational analysis of cell-to-cell heterogeneity in single-cell RNA-sequencing data reveals hidden subpopulations of cells. Nat. Biotechnol..

[5] Trapnell C (2015). Defining cell types and states with single-cell genomics. Genome Res..

[6] Huang S (2009). Non-genetic heterogeneity of cells in development: more than just noise. Development.

[7] Chang HH, Hemberg M, Barahona M, Ingber DE, Huang S (2008). Transcriptome-wide noise controls lineage choice in mammalian progenitor cells. Nature.

[8] Rocheteau P, Gayraud-Morel B, Siegl-Cachedenier I, Blasco MA, Tajbakhsh S (2012). A subpopulation of adult skeletal muscle stem cells retains all template DNA strands after cell division. Cell.

[9] Emery AE (2002). The muscular dystrophies. Lancet.

[10] Farup J, Madaro L, Puri PL, Mikkelsen UR (2015). Interactions between muscle stem cells, mesenchymal-derived cells and immune cells in muscle homeostasis, regeneration and disease. Cell Death Dis..

[11] Kharraz Y, Guerra J, Pessina P, Serrano LA, Munoz-Canoves P (2014). Understanding the process of fibrosis in Duchenne muscular dystrophy. Biomed. Res. Int..

[12] Uezumi A (2011). Fibrosis and adipogenesis originate from a common mesenchymal progenitor in skeletal muscle. J. Cell Sci..

[13] Vallecillo-García P (2017). Odd skipped-related 1 identifies a population of embryonic fibro-adipogenic progenitors regulating myogenesis during limb development. Nat. Commun..

[14] Uezumi A, Fukada SI, Yamamoto N, Takeda S, Tsuchida K (2010). Mesenchymal progenitors distinct from satellite cells contribute to ectopic fat cell formation in skeletal muscle. Nat. Cell Biol..

[15] Joe AW (2010). Muscle injury activates resident fibro/adipogenic progenitors that facilitate myogenesis. Nat. Cell Biol..

[16] Wosczyna MN (2019). Mesenchymal stromal cells are required for regeneration and homeostatic maintenance of skeletal muscle. Cell Rep..

[17] Heredia JE (2013). Type 2 innate signals stimulate fibro/adipogenic progenitors to facilitate muscle regeneration. Cell.

[18] Malecova B (2018). Dynamics of cellular states of fibro-adipogenic progenitors during myogenesis and muscular dystrophy. Nat. Commun..

[19] Marinkovic M (2019). Fibro-adipogenic progenitors of dystrophic mice are insensitive to NOTCH regulation of adipogenesis. Life Sci. Alliance.

[20] Reggio A (2020). Adipogenesis of skeletal muscle fibro/adipogenic progenitors is affected by the WNT5a/GSK3/β-catenin axis. Cell Death Differ..

[21] Lemos DR (2015). Nilotinib reduces muscle fibrosis in chronic muscle injury by promoting TNF-mediated apoptosis of fibro/adipogenic progenitors. Nat. Med..

[22] Amir ED (2013). viSNE enables visualization of high dimensional single-cell data and reveals phenotypic heterogeneity of leukemia. Nat. Biotechnol..

[23] Shannon P (2003). Cytoscape: a software environment for integrated models. Genome Res..

[24] Juban G (2018). AMPK activation regulates LTBP4-dependent TGF-β1 secretion by pro-inflammatory macrophages and controls fibrosis in Duchenne muscular dystrophy. Cell Rep..

[25] Uynuk-ool T (2017). The geometrical shape of mesenchymal stromal cells measured by quantitative shape descriptors is determined by the stiffness of the biomaterial and by cyclic tensile forces. J. Tissue Eng. Regen. Med..

[26] Carpenter AE (2006). CellProfiler: image analysis software for identifying and quantifying cell phenotypes. Genome Biol..

[27] Livak KJ, Schmittgen TD (2001). Analysis of relative gene expression data using real-time quantitative PCR and the 2^-ΔΔC^_T_ method. Methods.

[28] Dobin A (2013). STAR: ultrafast universal RNA-seq aligner. Bioinformatics.

[29] Anders S, Pyl PT, Huber W (2015). Genome analysis HTSeq—a Python framework to work with high-throughput sequencing data. Bioinformatics.

[30] Love MI, Huber W, Anders S (2014). Moderated estimation of fold change and dispersion for RNA-seq data with DESeq2. Genome Biol..

[31] Huang DW, Sherman BT, Lempicki RA (2009). Systematic and integrative analysis of large gene lists using DAVID bioinformatics resources. Nat. Protoc..

[32] Han H (2018). TRRUST v2: an expanded reference database of human and mouse transcriptional regulatory interactions. Nucleic Acids Res..

[33] Butler A, Hoffman P, Papalexi E, Satija R (2018). Integrating single-cell transcriptomic data across different conditions, technologies, and species. Nat. Biotechnol..

[34] Kuleshov MV (2016). Enrichr: a comprehensive gene set enrichment analysis web server 2016 update. Nucleic Acids Res..

[35] Dangain J, Vrbova G (1984). Muscle development in mdx mutant mice. Muscle Nerve.

[36] Pastoret C, Sebille A (1995). mdx mice show progressive weakness and muscle deterioration with age. J. Neurol. Sci..

[37] Kobayashi T (2009). The cyclic gene Hes1 contributes to diverse differentiation responses of embryonic stem cells. Genes Dev..

[38] Mills JC, Stanger BZ, Sander M (2019). Nomenclature for cellular plasticity: are terms as plastic as the cells themselves?. EMBO J..

[39] Bruyère C (2019). Actomyosin contractility scales with myoblast elongation and enhances differentiation through YAP nuclear export. Sci. Rep..

[40] Merrick D (2019). Identification of a mesenchymal progenitor cell hierarchy in adipose tissue. Science.

[41] Kopinke D, Roberson EC, Reiter JF (2017). Ciliary Hedgehog signaling restricts injury-induced adipogenesis. Cell.

[42] Cerquone Perpetuini A (2020). Janus effect of glucocorticoids on differentiation of muscle fibro/adipogenic progenitors. Sci. Rep..

[43] Thonberg H, Fredriksson M, Nedergaard J, Cannon B (2002). A novel pathway for adrenergic stimulation of cAMP-response-element-binding protein (CREB) phosphorylation: mediation via α1-adrenoceptors and protein kinase C activation. Biochem. J..

[44] Reusch JE, Colton LA, Klemm DJ (2000). CREB activation induces adipogenesis in 3T3-L1 cells. Mol. Cell Biol..

[45] Hinz B (2007). The myofibroblast: one function, multiple origins. Am. J. Pathol..

[46] Contreras O (2019). Cross-talk between TGF-β and PDGFRα signaling pathways regulates the fate of stromal fibro-adipogenic progenitors. J. Cell Sci..

[47] Wang C (2007). Adiponectin sensitizes insulin signaling by reducing p70 S6 kinasemediated serine phosphorylation of IRS-1. J. Biol. Chem..

[48] Ryu J (2014). APPL1 potentiates insulin sensitivity by facilitating the binding of IRS1/2 to the insulin receptor. Cell. Rep..

[49] Okada-Iwabu M (2013). A small-molecule AdipoR agonist for type 2 diabetes and short life in obesity. Nature.

[50] Kafadar KA (2009). Sca-1 expression is required for efficient remodeling of the extracellular matrix during skeletal muscle regeneration. Dev. Biol..

[51] The Tabula Muris Consortium. (2020). A single-cell transcriptomic atlas characterizes ageing tissues in the mouse. Nature.

[52] Palma A (2019). Myo-REG: a portal for signaling interactions in muscle regeneration. Front. Physiol..

[53] Lukjanenko L (2019). Aging disrupts muscle stem cell function by impairing matricellular WISP1 secretion from fibro-adipogenic progenitors. Cell Stem Cell.

[54] Spencer SL, Gaudet S, Albeck JG, Burke JM, Sorger PK (2009). Non-genetic origins of cell-to-cell variability in TRAIL-induced apoptosis. Nature.

[55] Dietrich JE, Hiiragi T (2007). Stochastic patterning in the mouse pre-implantation embryo. Development.

[56] Morris SA (2019). The evolving concept of cell identity in the single cell era. Development.

[57] Tonlorenzi, R., Dellavalle, A., Schnapp, E., Cossu, G. & Sampaolesi, M. Isolation and characterization of mesoangioblasts from mouse, dog, and human tissues. *Curr. Protoc. Stem Cell Biol.* 2007; Cahpter 2: Unit 2B.1. 10.1002/9780470151808.sc02b01s3 18785178

[58] Mitchell KJ (2010). Identification and characterization of a non-satellite cell muscle resident progenitor during postnatal development. Nat. Cell Biol..

[59] Liu N (2017). Twist2-dependent progenitor cell contributes to adult skeletal muscle. Nat. Cell Biol..

[60] Palumbo R (2004). Extracellular HMGB1, a signal of tissue damage, induces mesoangioblast migration and proliferation. J. Cell Biol..

[61] Camps J (2020). Interstitial cell remodeling promotes aberrant adipogenesis in dystrophic muscles. Cell Rep..

[62] Birbrair A (2013). Skeletal muscle pericyte subtypes differ in their differentiation potential. Stem Cell Res..

[63] Altschuler SJ, Wu LF (2010). Cellular heterogeneity: do differences make a difference?. Cell.

[64] Mojtahedi M (2016). Cell fate decision as high-dimensional critical state transition. PLoS Biol..

[65] Bulfield G, Siller WG, Wight PAL, Moore KJ (1984). X chromosome-linked muscular dystrophy (mdx) in the mouse. Proc. Natl Acad. Sci..

[66] Loewer A, Lahav G (2011). We are all individuals: causes and consequences of non-genetic heterogeneity in mammalian cells. Curr. Opin. Genet. Dev..

