# Supplementary material for: SCA-1 micro-heterogeneity in the fate decision of dystrophic fibro/adipogenic progenitors
Source: Cell Death Dis. 2021 Jan 25;12(1):122. doi: 10.1038/s41419-021-03408-1 (PMC7835386; doi:10.1038/s41419-021-03408-1)
Supplement: Supplementary file 1 — Supplementary Information [file 41419_2021_3408_MOESM1_ESM.docx]

**SCA-1 micro-heterogeneity in the fate decision of dystrophic fibro/adipogenic progenitors**

Running title: FAP micro-heterogeneity in dystrophic mice

Giulio Giuliani^1,^ *, Simone Vumbaca^1^, Claudia Fuoco^1^, Cesare Gargioli^1^, Ezio Giorda^2^, Giorgia Massacci^1^, Alessandro Palma^1^, Alessio Reggio^1^, Federica Riccio^1^, Marco Rosina^1^, Maria Vinci^3^, Luisa Castagnoli^1^ and Gianni Cesareni^1,4^

^1^ Department of Biology, University of Rome “Tor Vergata”, Rome, Italy

^2^ Core Facilities, Bambino Gesù Children’s Hospital – IRCCS, Rome, Italy

^3^ Department of Onco-haematology, Cell and Gene Therapy, Bambino Gesù Children’s Hospital – IRCCS, Rome, Italy

^4^ Fondazione Santa Lucia Istituto di Ricovero e Cura a Carattere Scientifico (IRCCS), Rome, Italy

*corresponding author, email: [g.giulianigiulio@gmail.com](mailto:g.giulianigiulio@gmail.com)

**Supplementary Information**

**Analysis of the antigen expression data from the multiplex flow cytometry assay**

In order to identify FAPs among the mononuclear cell populations of the skeletal muscle, we reduced the dimensionality of our data by applying the t-distributed stochastic neighbour embedding (tSNE) algorithm. Density plots of the resulting viSNE maps highlight a redistribution of the abundance of cell populations in *mdx* mice when compared to wild type (Supplementary Fig. 1a), suggesting a modulation of the profile of muscle mononuclear cell populations in the two mouse models. Next, we applied the FlowSOM algorithm to identify 25 metaclusters of cells with distinct expression profiles and we mapped these metaclusters onto the viSNE maps to facilitate result interpretation (Supplementary Fig. 1b). Finally, we scrutinized the antigens expressed in the different metaclusters by consulting the viSNE maps (Supplementary Fig. 1c) and we collapsed the metaclusters, showing comparable antigen expression patterns, into new larger clusters.

**Supplementary figure legends**

**Supplementary Fig. 1 Identification of fibro/adipogenic progenitors among the total mononuclear cells. a** Density representation of viSNE maps showing a redistribution of density patterns between wild type and *mdx* mice. Colour scale ranges from black (low density) to yellow (high density). Each plot represents a biological replicate. **b** Representative viSNE maps illustrating the 25 metaclusters created by the FlowSOM algorithm in wild type and *mdx* mice. These metaclusters were mapped onto the viSNE maps to facilitate the interpretation of the results. Each metacluster is indicated by a different colour. **c** viSNE maps showing the expressing of antigens used in the clustering. These viSNE maps were used to identify metaclusters having similar expression profiles (this is the expert manual refinement phase of our method). The result of such approach are the four clusters in Fig. 1b (the four main muscle populations) and 11 unassigned clusters in Supplementary Fig. 1d. **d** Representative viSNE maps showing the 11 unassigned clusters. **e** Bar plot indicating the percentage of fibro/adipogenic progenitors (FAPs) in wild type and *mdx* mice. Data are presented as mean ± SEM. Statistical significance was estimated by a Student t-test, * p ≤ 0.05 (n = 4).

**Supplementary fig. 2 Self-organising maps (SOMs) representing expression variability of surface antigens in fibro/adipogenic progenitors.** The expression of CD34, CD146, CD90.2, CD140b, CD140a and SCA-1 in FAPs (identified through the approach described in Supplementary Fig. 1) were analysed by the FlowSOM algorithm. FlowSOM output are the self organising maps (SOMs) showed in this Supplementary Figure. Each node represents a group of cells (clusters). Nodes with similar expression patterns were grouped into 4 metaclusters indicated by their outline colour: black, blue, green and red. Node size is proportional to the number of cells in each cluster. Node filling colour represents the expression level of the antigen ranging from low level (blue) to high level (red). SOM showing SCA-1 expression is reported in Fig. 1c.

**Supplementary Fig. 3 Sorting strategy to isolate fibro/adipogenic progenitors from *mdx* mice. a** Representative dot plots showing the sorting strategy to isolate FAP cell states from hind limb muscles of *mdx* mice. CD45^-^ CD31^-^ cells were isolated by magnetic activated cell sorting (MACS). Next, FAP cell states were isolated by fluorescent activated cell sorting (FACS) as Integrin alpha-7^-^ SCA-1^+^ cells. MuSCs were isolated as Integrin alpha-7^+^ cells. **b** Fluorescence minus one (FMO) controls used to place gates of the sorting strategy. **c** Purity of FAP cell states isolated from *mdx* mice. **d** Representative images of FAP cell states from *mdx* mice immunolabelled for CD140a (green). Nuclei were counterstained with Hoechst 33342 (blue). Scale bar 100 μm. **e** Flow cytometry analysis of CD140a expression in FAP cell states expanded in Cytogrow. From left to right: histograms of FMO controls and histograms of stained cells. **f** bar plot illustrating the percentage of FAP cell states positive for CD140a. Data were presented as mean ± SEM. (n = 3). **g** NBT/BCIP assay to evaluate alkaline phosphatase activity in FAP cell states. Nuclei were counter stained with Hoechst 33342. **h** Immunofluorescence against myosin heavy chain (MyHC) in FAP cell states.

**Supplementary fig. 4 SCA1-High FAPs and SCA1-Low-FAPs are two dynamic cell states and share the same morphology. a** and **b** report representative gating strategy applied to obtain SCA-1 histogram of FAPs in Fig. 2a. In **a** are reported contour plots of unstained CD45^-^ CD31^-^ cells. In **b** are reported contour plots of CD45^-^ CD31^-^ cells stained with antibodies against Integrin alpha-7 and SCA-1. CD45^-^ CD31^-^ cells were isolated by MACS from hind limb muscles of *mdx* mice. **c** FMO controls and unstained cells of samples in **d**, **e** and Fig. 2a. **d** and **e** show gating strategies to study SCA-1 expression in SCA1-High-FAPs and SCA1-Low-FAPs, respectively. Moreover, here we demonstrate that these cells express also CD140a. **f** Aspect ratio (AR) and roundness values changing from a round shape to an elongated shape. **g** and **h** are the distributions of AR and roundness values in *mdx* MuSCs (n = 181) cultured *ex vivo* and stained using CFSE. These values derived from three different biological replicates. Distributions highlight different peaks corresponding to round myoblasts (high roundness/ low AR) and elongated myocytes (low roundness/ high AR). **i** Box plots reporting the CFSE positive area expressed in μm^2^ of SCA1-High-FAPs (n = 96), SCA1-Low-FAPs (n = 94) and MuSCs (n = 181). Whiskers are minimum and maximum values of the distributions. **j** Crop of representative images of round (left) and elongated (right) *mdx* MuSCs stained with CFSE (yellow) and counterstained with Hoechst 33342 (blue). Scale bar 50 μm. Statistical significance was estimated by a two tailed Mann-Whitney test after a normality test. **** p < 0.0001

**Supplementary fig. 5 Nuclei quantification of FAP cell states induced to differentiate into adipocytes and myofibroblasts. a** Bar plot reporting nuclei quantification per filed of the experiment in Fig. 3b-c (n = 4). **b** Bar plot representing the number of nuclei per filed of the experiment in Fig. 3f-g (n = 3). Statistical analysis was performed using Two-way ANOVA in **a** and two tailed Student t-test in **b**. **c** Dot plot representing the number of nuclei over the number of adipocytes in each field for SCA1-High-FAPs (in red) and SCA1-Low-FAPs (in blue) from the experiment in Fig. 3a-d. Trend lines were calculated using GraphPad Prism 7 software. **d** Bar plots showing SCA-1 expression (in arbitrary units) in bulk FAPs and FAP cell states investigated by mass cytometry. **e** Bar plot of nuclei per field of the experiment in Fig. 4b (n = 6). Statistical analysis was evaluated using Two-way ANOVA. Data are presented as mean ± SEM. Data are presented as mean ± SEM. * p< 0.05; ** p < 0.01.

**Supplementary fig. 6 Adiponectin mediates a possible autocrine signal that promotes adipogenesis of SCA1-High-FAPs. a** Real-time PCR analysis of *Adipoq* mRNA in FAP cell states after 5 days in GM (n = 4). **b** A simplified network of the adiponectin pathway. Red nodes are protein of the pathway whose mRNA are upregulated in SCA1-High-FAPs. **c** Representative images of experiment in Supplementary fig.6d. FAP cell states were treated for 24 hours with either 25 μM AdipoRon or DMSO and next adipogenic differentiation was induced with the described protocol. **d** Bar plot showing the percentage of ORO positive (n = 2). **e**-**h** Bar plots showing the normalised expression of *Mmp11*, *Mmp23*, *Mmp19* and *Alpl* mRNAs from RNA-seq data. Data are presented as mean ± SEM. Data are presented as mean ± SEM. * p< 0.05.

**Supplementary fig. 7 *In vitro* and *in vivo* proliferation of FAP cell states. a** Nonlinear regression curves of T0, 24 h and 48 h time points of the growth curves in Fig. 6b. These curves and doubling times were calculated using the Exponential growth equation tool of Graph Pad Prism. Data are presented as mean ± SEM (n = 4). **b** and **c** are respectively FMO controls and unstained cells of samples in Fig. 6g.

**Supplementary fig. 8 Analysis at single-cell level of FAP cell states in wild type mice taking advantage of the Tabula Muris Senis dataset. a** Table reporting the biomarkers used to assign a cell line ontology ID to each cluster. **b**-**l** Violin plots displaying the distribution of each biomarker across the different clusters for all the identified cell populations.

**Supplementary table legends**

**Supplementary table 1**

Full list of the antibodies applied in the multiplex flow cytometry assay.

**Supplementary table 2**

Full list if the antibodies applied in the mass cytometry experiment.

**Supplementary table 3**

Real-time PCR primers.

**Supplementary table 4**

Biological Process GO terms enriched in up regulated and downregulated genes of SCA1-Low-FAPs.
