# Supplementary figures and images for: SCA-1 micro-heterogeneity in the fate decision of dystrophic fibro/adipogenic progenitors
Source: Cell Death Dis. 2021 Jan 25;12(1):122. doi: 10.1038/s41419-021-03408-1 (PMC7835386; doi:10.1038/s41419-021-03408-1)

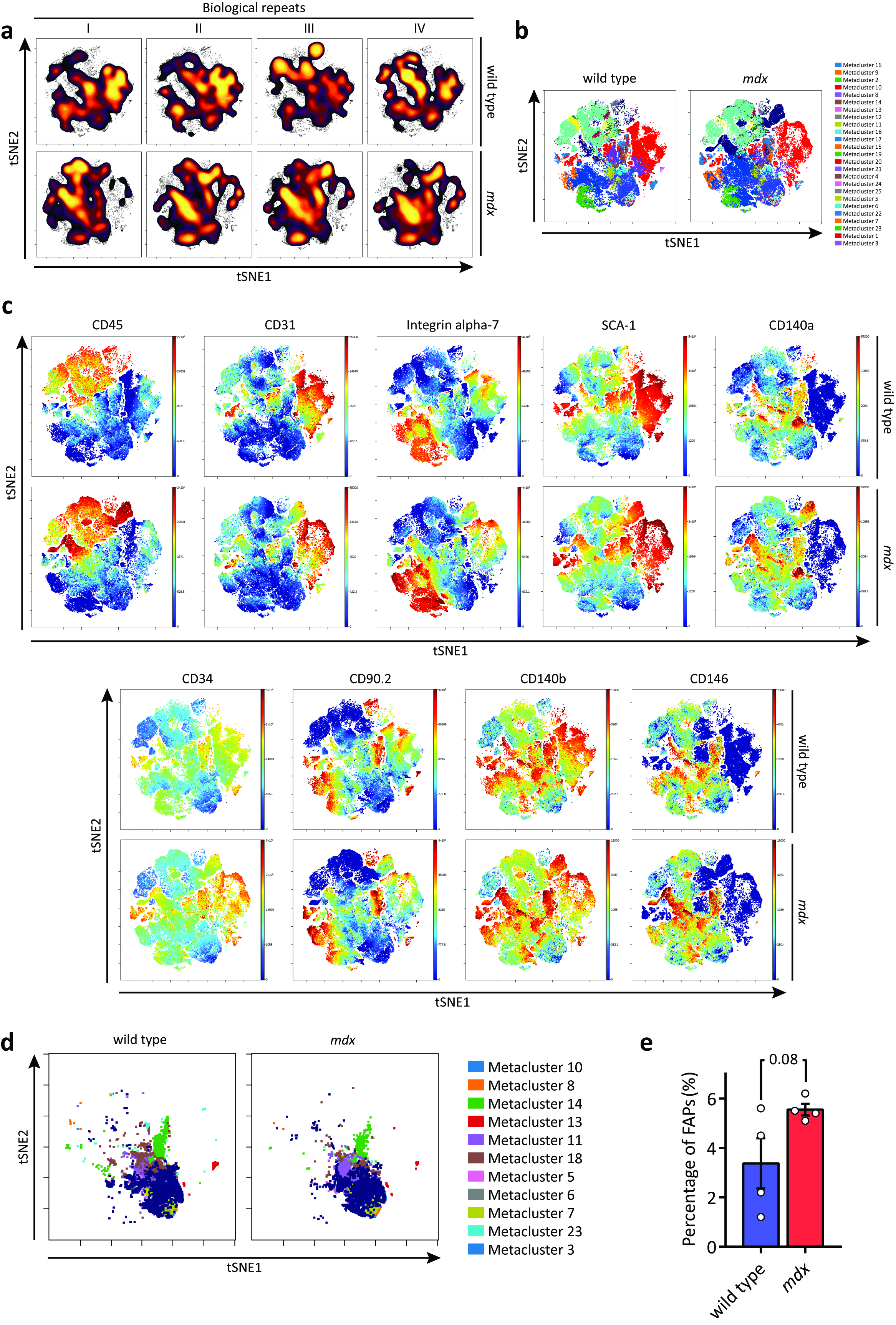

Supplement: Supplementary file 6 — Supplementary figure 1 [file 41419_2021_3408_MOESM6_ESM.tif]

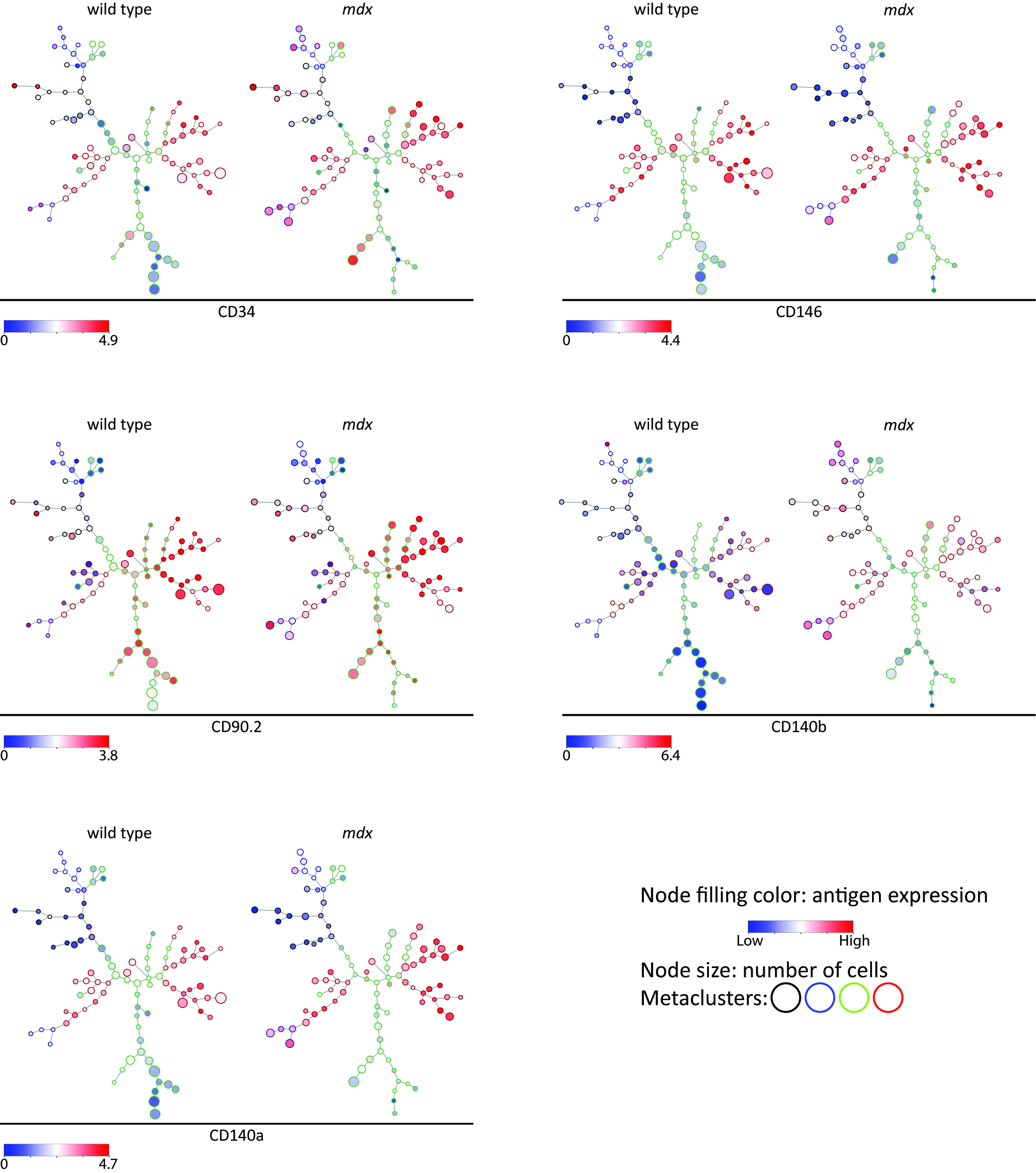

Supplement: Supplementary file 7 — Supplementary figure 2 [file 41419_2021_3408_MOESM7_ESM.tif]

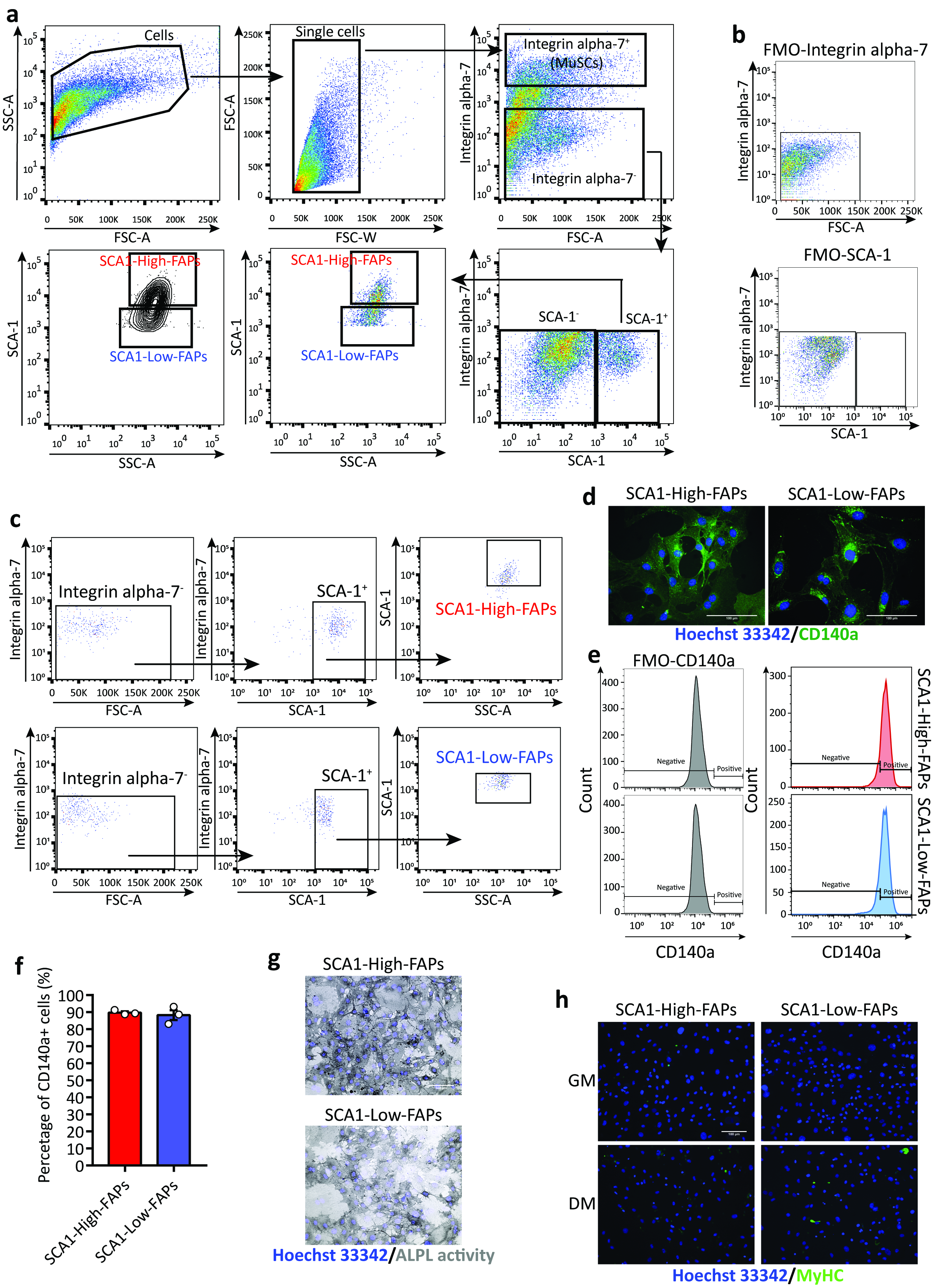

Supplement: Supplementary file 8 — Supplementary figure 3 [file 41419_2021_3408_MOESM8_ESM.tif]

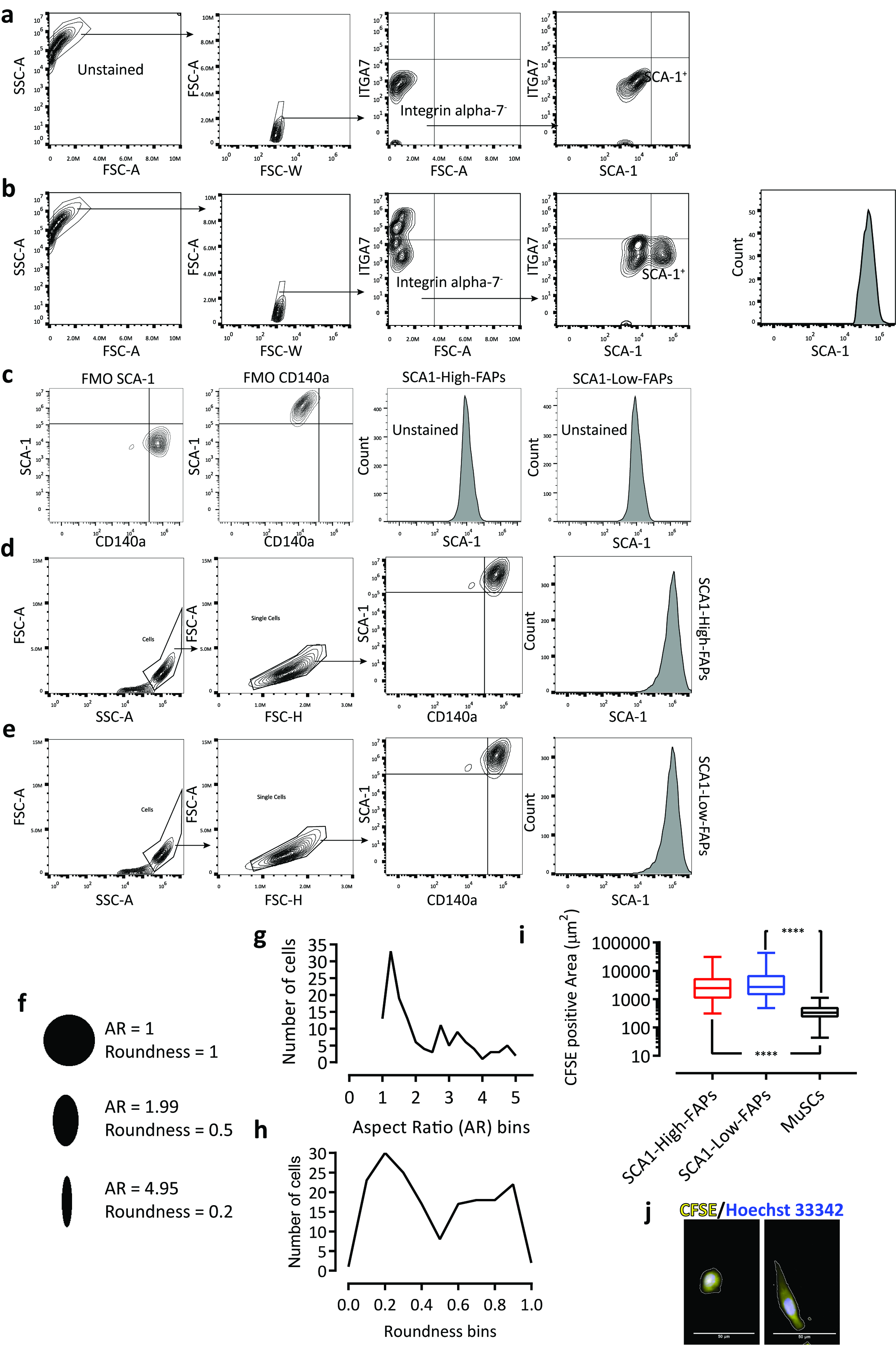

Supplement: Supplementary file 9 — Supplementary figure 4 [file 41419_2021_3408_MOESM9_ESM.tif]

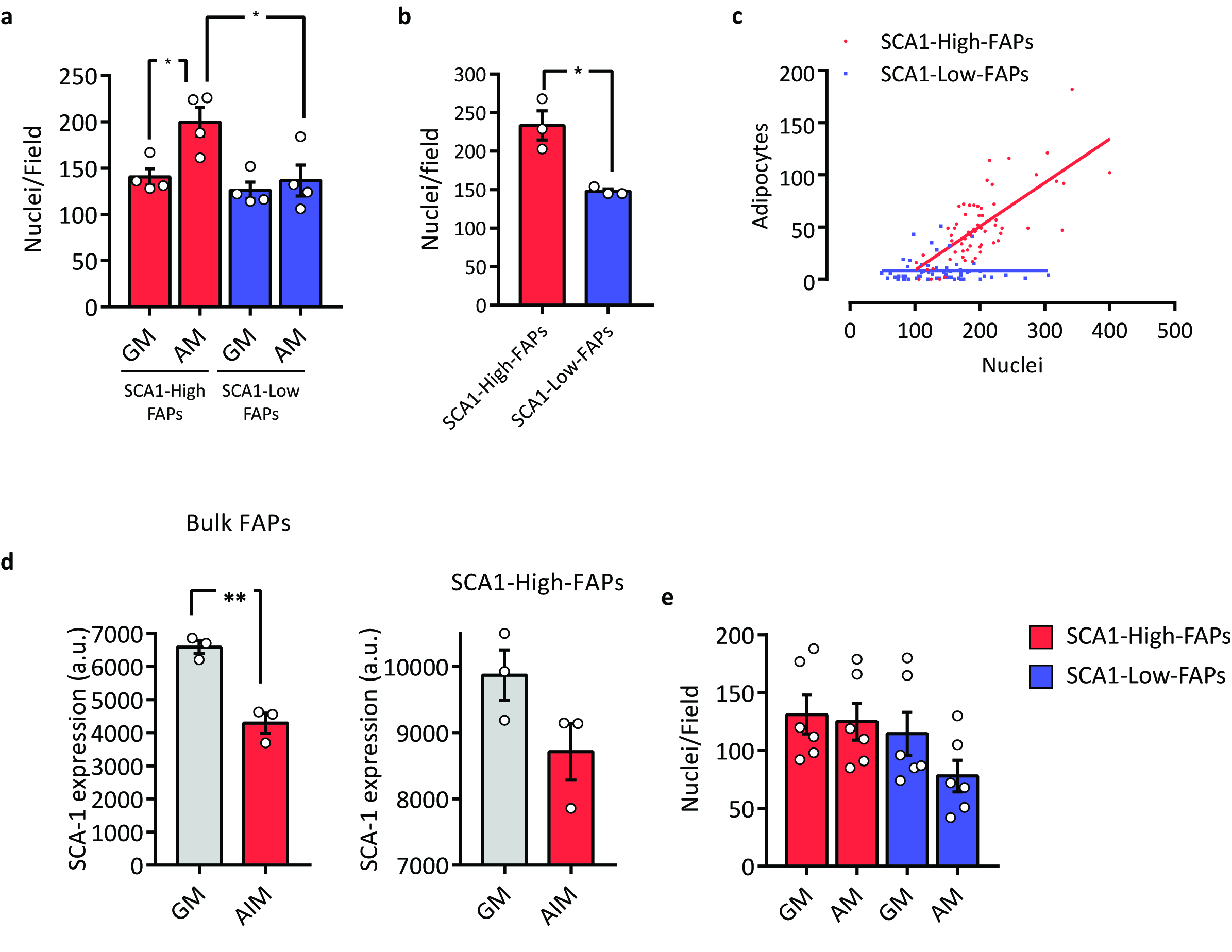

Supplement: Supplementary file 10 — Supplementary figure 5 [file 41419_2021_3408_MOESM10_ESM.tif]

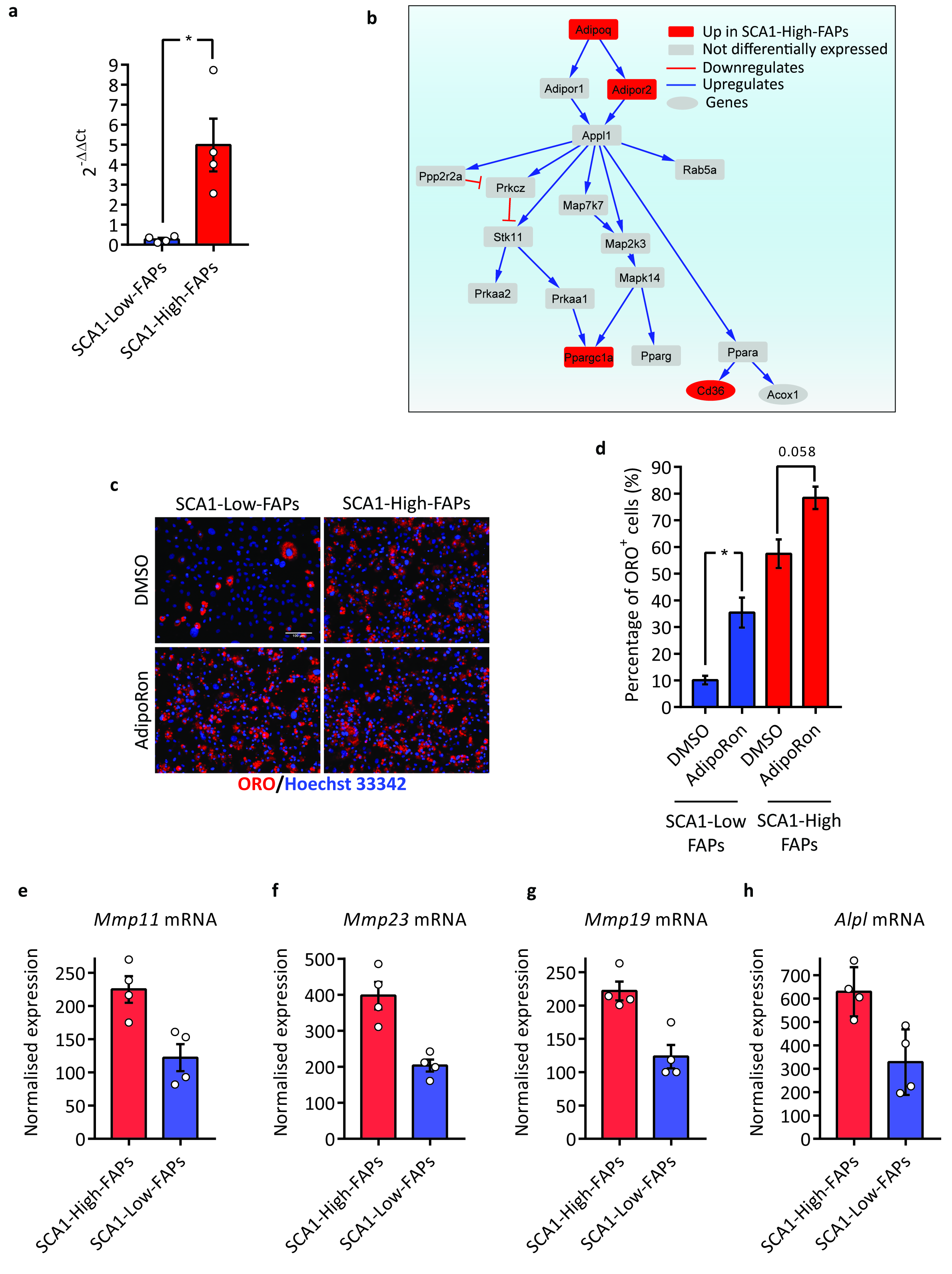

Supplement: Supplementary file 11 — Supplementary figure 6 [file 41419_2021_3408_MOESM11_ESM.tif]

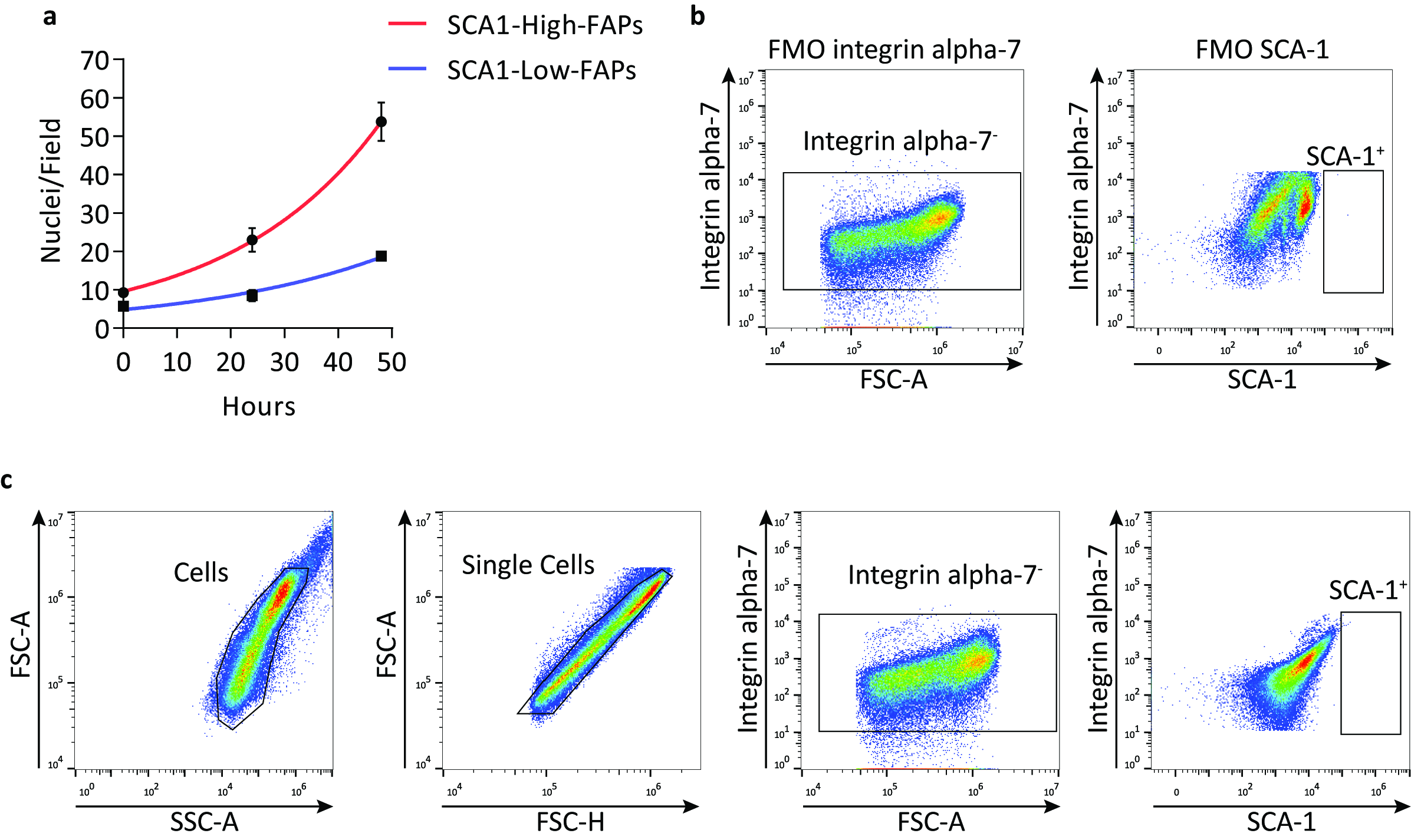

Supplement: Supplementary file 12 — Supplementary figure 7 [file 41419_2021_3408_MOESM12_ESM.tif]

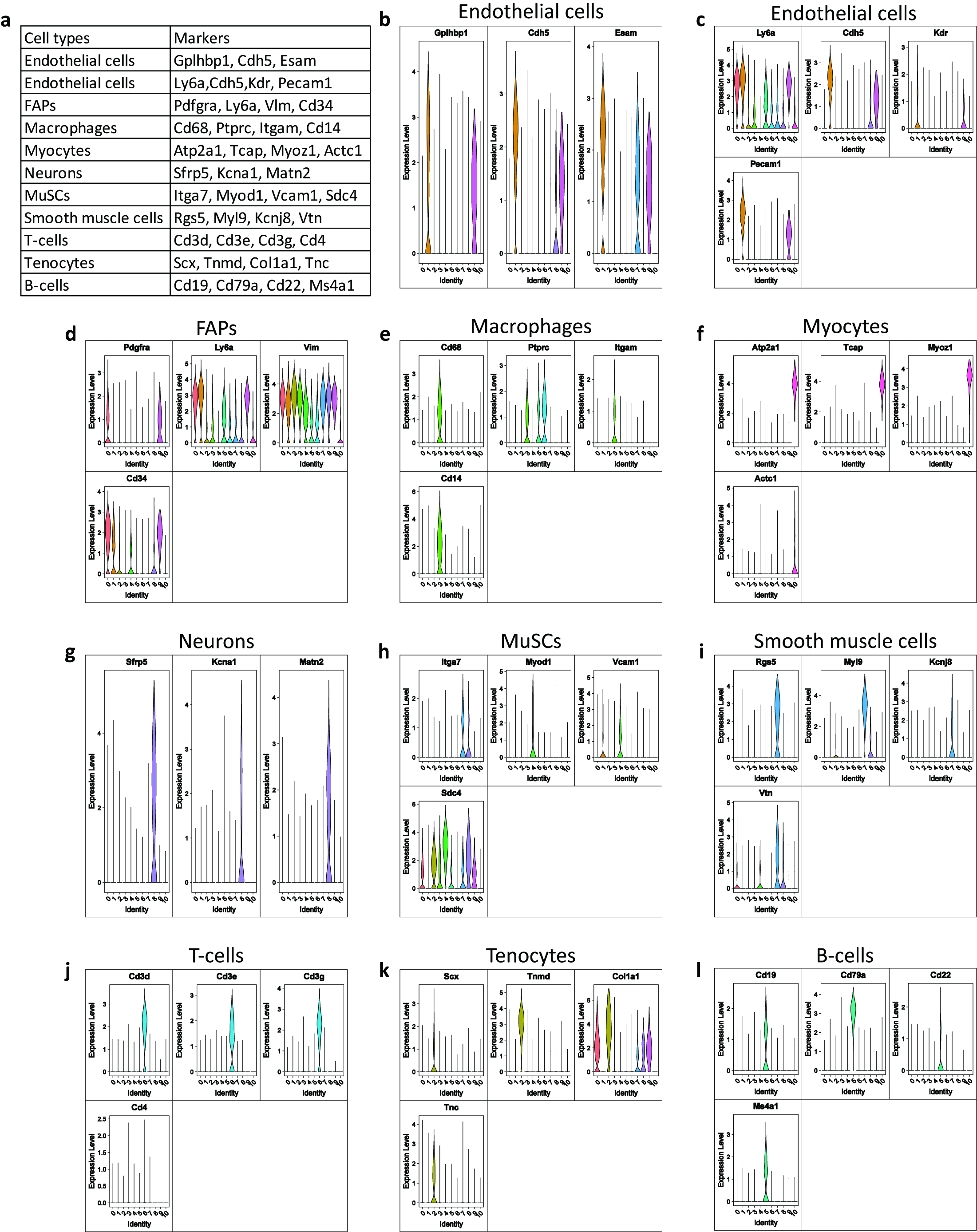

Supplement: Supplementary file 13 — Supplementary figure 8 [file 41419_2021_3408_MOESM13_ESM.tif]
